# Distinct changes in tomato-associated multi-kingdom microbiomes during *Meloidogyne incognita* parasitism

**DOI:** 10.1186/s40793-024-00597-y

**Published:** 2024-07-27

**Authors:** Enoch Narh Kudjordjie, Susana S Santos, Olivera Topalović, Mette Vestergård

**Affiliations:** 1 Department of Agroecology, Faculty of Technical Sciences, Aarhus University, Slagelse, 4200 Denmark; 2 Department of Biology, Section of Terrestrial Ecology, University of Copenhagen, Copenhagen, 2100 Denmark

**Keywords:** Plant parasitic nematodes, Plant-associated microbiomes, Tripartite networks, Time-series analysis, Correlation analysis

## Abstract

**Background:**

The interplay between root-knot nematode (RKN) parasitism and the complex web of host-associated microbiota has been recognized as pivotal for effective management of the pest. However, studies assessing this relationship have focussed on the bacterial and fungal communities, neglecting the unicellular eukaryotic members. Here, we employed amplicon sequencing analysis of the bacterial 16S rRNA, fungal ITS and eukaryotic 18S rRNA genes, and comprehensively examined how the microbiome composition, diversity and networking developed with time in the rhizospheres and roots of RKN-inoculated and non-inoculated tomato plants.

**Results:**

As expected, infection with the RKN *Meloidogyne incognita* decreased plant growth. At individual timepoints, we found distinct bacterial, fungal and eukaryote community structures in the RKN-inoculated and non-inoculated rhizospheres and roots, and RKN inoculation affected several taxa in the root-associated microbiome differentially. Correlation analysis revealed several bacterial and fungal and few protist taxa that correlated negatively or positively with *M. incognita*. Moreover, network analysis using bacterial, fungal and eukaryotic data revealed more dynamic networks with higher robustness to disturbances in the RKN-inoculated than in the non-inoculated rhizospheres/roots. Hub taxa displayed a noticeable successional pattern that coincided with different phases of *M. incognita* parasitism. We found that fungal hubs had strong negative correlations with bacteria and eukaryotes, while positive correlations characterized hub members within individual kingdoms.

**Conclusion:**

Our results reveal dynamic tomato-associated microbiomes that develop along different trajectories in plants suffering *M. incognita* infestation and non-infested plants. Overall, the results identify stronger associations between RKN and bacterial and fungal taxa than between eukaryotic taxa and RKN, suggesting that fungal and bacterial communities could play a larger role in the regulation of RKN. The study identifies several putative RKN-antagonistic bacterial and fungal taxa and confirms the antagonistic potential previously identified in other taxa.

**Supplementary Information:**

The online version contains supplementary material available at 10.1186/s40793-024-00597-y.

## Introduction

Plant parasitic nematodes including root-knot nematodes (RKN), *Meloidogyne* spp. are major threats to crop production worldwide, causing high financial costs in the production of numerous important crops, such as soybean, potatoes, tomatoes, or carrots [1]. Efficient and sustainable management of RKN will require a detailed examination of the factors, specifically, within the host ecological niche that influence disease development and progression. Recent studies have revealed a complex web of interaction between the invading pest and the plethora of microorganisms associated with the plant, collectively called the microbiome [2–5]. The host associated microbiota including bacteria, fungi and protists play an important role promoting plant growth, e.g. via nutrient acquisition, disease suppression, and induction of tolerance to abiotic stresses [6, 7]. The host-associated microbiota’s ability to suppress invading pathogens or pests has emerged as a focal point for sustainable control strategies.

RKN are microscopic, obligate endoparasites that attack a broad range of plants. RKN infection starts with second-stage juveniles (J2s) that migrate into the plant roots, becoming parasitic by initiating feeding sites within the vascular cylinder to complete their life cycle (usually 4–6 weeks) [8]. These feeding sites result in galls made up of multiple multinucleate giant cells that can be easily recognized. Infection can impair root functioning and cause plant death if accompanied by other stressors, e.g. low soil nutrient availability and water deficit [9]. RKN infection of a host plant is accompanied by RKN secretion of molecules aiding parasitism and the activation of host defense compounds [10]. The infection and disease development follows time-dependent distinct phases involving both physical and molecular mechanisms that hijack the host regulatory and metabolic machinery [10]. Shukla et al., (2018) revealed a detailed molecular arsenal of *M. incognita* and tomato defensive molecules that characterize the different parasitic phases of infection.

Increasing evidence shows both direct and indirect effects of the host-associated microbiota on RKN during invasion and, vice versa, RKN attack affects microbial communities in plant compartments [2, 11]. Studies on impacts of the soil microbiome on RKN activity revealed that non-RKN-infested soils had higher microbial diversity than RKN-infested soils, with bacteria such as *Pseudomonas* sp. and *Bacillus* sp. screened as potential biocontrol agents [5, 12]. Beneficial microbes antagonize different stages of plant-parasitic nematodes, especially eggs and the infective stages in soil directly via antibiosis (via secondary metabolites and lytic enzymes), parasitism, and paralysis [13, 14]. Some rhizobacteria including *Rhizobium* spp., *Burkholderia* spp., *Pseudomonas* spp., *Bacillus* spp., and fungi such as *Trichoderma* spp. are known to interfere with nematode-host recognition, nematode behaviour, feeding and reproduction, and induce systemic resistance in plants [13–17]. Moreover, microbes attached to infective stages of nematodes in soil have been suggested to be involved in soil suppressiveness towards them, while other microorganisms may protect nematodes against microbial suppression [4, 18, 19]. Therefore, understanding pathobiotic systems, including their structure and functions is a prerequisite for understanding the pathogenesis, persistence and suppressiveness of RKN.

Microbial communities are characterized by extensive and dynamic inter- and intra-kingdom interactions [20, 21], specifically during pathogen invasion [21, 22]. Network-based approaches are widely used to explore these complex interactions, with strong correlations found among prokaryotes and eukaryotic taxa [23]. Network analysis enable us to resolve ecologically important taxa such as indicator species or hub members in microbial communities [20, 24]. Hub taxa are those with many network connections, and their removal could disrupt the overall ecological network structure [24]. A tripartite analysis integrating bacteria, fungal and protist data revealed protists as key hubs connecting bacterial and fungal communities [25]. Nonetheless, such analyses are limited and thus, studies are needed to disentangle these connections. Although specific microbes affect RKNs, there is limited knowledge on their interaction with the entire host associated microbiome (i.e. bacteria, fungi and protists). Moreover, because RKN infection is characterized with distinct parasitic phases, a detailed understanding of how the plant microbiome alters during RKN parasitism would provide new mechanistic insights into the complex configurations associated with nematode attack of susceptible hosts. Therefore, we characterized the multi-trophic inter-kingdom microbiome associated with *M. incognita* infection in tomato plants, along time, under greenhouse conditions.

We hypothesize that RKN infection causes time-dependent community shifts that affect the microbiome co-occurrence patterns and overall network stability. The objectives of this study were to (i) compare bacterial, fungal and micro-eukaryotic (predominantly protists and nematode) communities in bulk, rhizosphere and root compartments of RKN-inoculated and non-inoculated tomato plants at different growth stages; (ii) identify microbial and eukaryotic taxa that are differentially affected by RKN infection and putatively involved in RKN regulation; (iii) examine the multi-kingdom community interactions between bacterial, fungal, protist and nematodes in RKN inoculated and non-inoculated rhizosphere/roots of tomato.

## Materials and methods

### Plant material and root-knot nematode inoculum preparation

We used the tomato cultivar *Solanum lycopersicum* cv. Moneymaker, which is susceptible to *M. incognita*. The seeds were surface sterilized with 1.5% sodium hypochlorite for 15 min and then rinsed 5 times with sterile deionized water. The seeds were germinated for 3 days on sterile paper tissues. Seedlings were then planted into potting soil and kept in a greenhouse (3 weeks, 16 h photoperiod, at 25 °C).

The root knot nematode *M. incognita* was multiplied on tomato cv. Moneymaker for 2 months in the greenhouse at 16 h photoperiod at 25 °C. The J2s were collected by macerating nematode infested roots in 0.5% sodium hypochlorite for 30 s in a commercial blender. The macerate was washed with tap water and passed through a 500 μm nested over a 100 μm and 20 μm sieve. Plant debris collected on the upper sieves were discarded and eggs were collected and transferred to a modified Baermann tray with tap water to facilitate egg hatching. We collected hatched J2s daily for 4 days. The collected J2 cultures were surface sterilized: Nematodes were placed on 5 μm sieves (Cell-Trics1 filters, Sysmex, Norderstedt, Germany) and washed with 10 ml sterilized tap water. Following, we incubated the nematodes for 4 h in 5 ml 1x CellCultureGuard (AppliChem, Darmstadt, Germany) on a rotary shaker at 150 rpm. Finally, the nematodes were washed on a 5 μm sieve and incubated overnight in sterilized tap water. Prior to use in the experiments, nematodes were checked for their sterility by plating a subsample of them on LB plates for bacterial growth for 8 days.

### Greenhouse experiment

The experimental approach is outlined in Supplementary figure S1. Soil (sandy clay soil, pH 5.9) was collected from an organically managed field in Skælskør, Denmark, in February 2019. The field was previously planted with Faba beans. The soil was homogenized, mixed with potting soil (1:1 v/v) and transferred into 2 L planting pots. Uniformly developed 2-week-old seedlings were planted in each pot. Tomato plants were left to grow for one week in the soil mixture prior to nematode inoculation. Pots with soil mixture but without plants were used as bulk soil and treated as the planted pots.

We inoculated half of the planted pots with infective *M. incognita* stage J2 by transferring 1 ml of a J2 suspension with 1000 J2s/ml into 4 holes around the plant (4000 per pot). Each treatment was replicated six times with a pot representing a replicate, for each sampling time (0, 3, 7, 30 and 60 days post inoculation (dpi)). We collected a total of 180 soil and root samples (i.e. 2 treatments (J2 inoculated and non-inoculated) x 5 sampling times x 6 replicates x 3 compartments (bulk soil (BK), rhizosphere soil (RS), roots (RTS)). During the experiment, we kept the plants in the greenhouse at 25 °C with 16 h of light and watered and fertilized once a week.

### Plant growth parameters and soil and root sampling

**Fig. 1:**
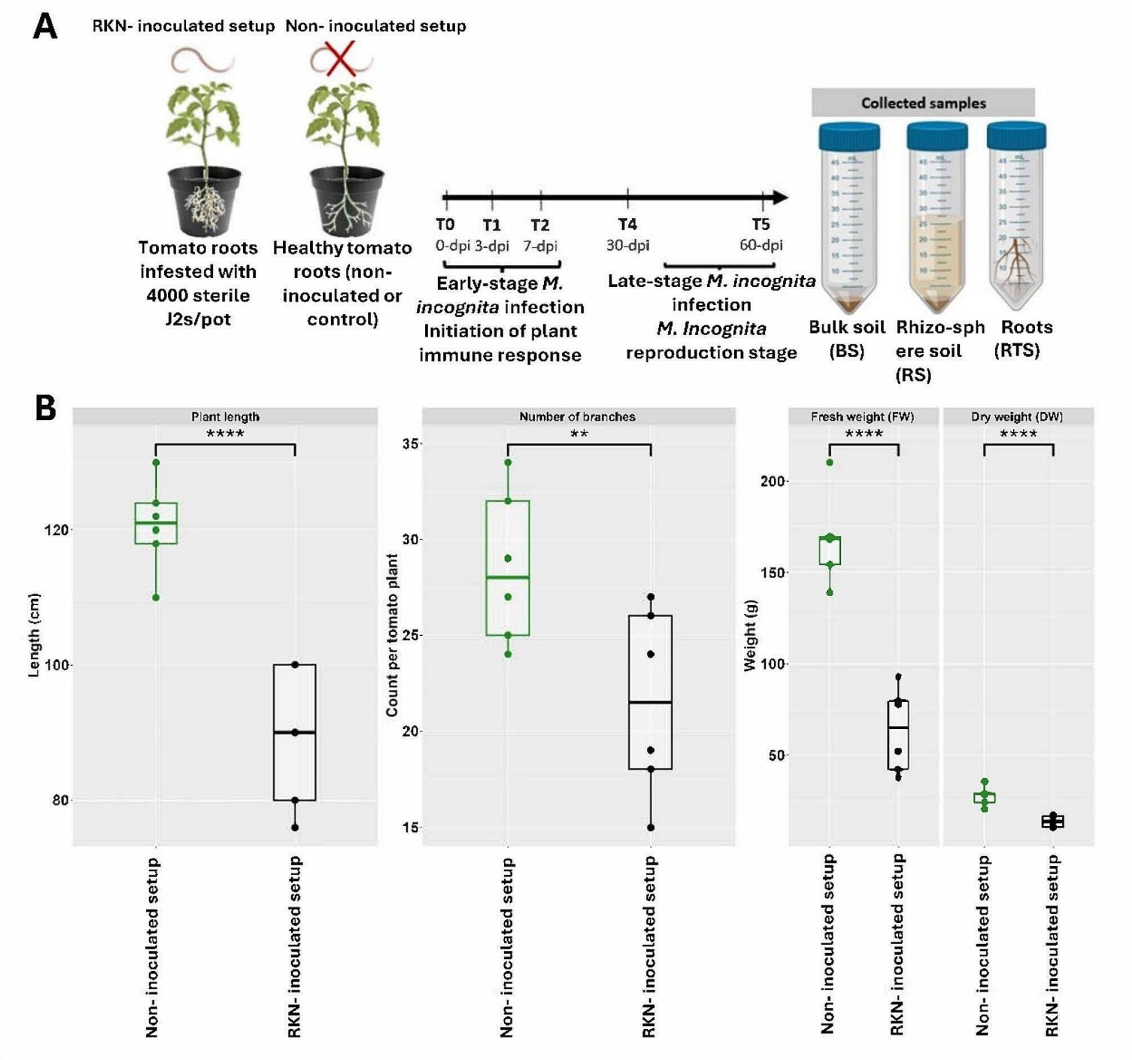
(**A**) Experimental timeline and sampling scheme. The timeline from the first day of RKN inoculation to the last sampling at 60 days post inoculation (dpi). Early time points (0, 3 and 7 dpi) were assumed to coincide with *M. incognita* infection initiation and plant immune defense activation, and late-stage post RKN root penetration (30 and 60) including the reproductive phase. (**B**) Plant growth parameters (plant length, number of branches, shoot fresh weight (FW) and dry weight (DW) of RKN-inoculated and non-inoculated tomato plants at 60 dpi

We measured plant height, number of branches, shoot fresh and shoot dry weight at each sampling time (Fig. 1A, Supplementary Table S1). The complete above-ground part of the plant was harvested for fresh and dry biomass determination. Soil and plant samples were collected at the site of plant growth (greenhouse) to prevent changing environmental conditions that impact microbial community composition associated with plant organs [26]. At each sampling time, we removed the plants from the pots and liberated the root system from the soil by shaking. To separate the RS from the roots, we vortexed the roots with closely adhering RS in a 50 ml tube. Two g of RS were immediately frozen in liquid N_2_ and stored at -80 °C for downstream molecular analysis. Likewise, the RTS were immediately frozen in liquid N_2_ and stored at -80 °C. From BK pots, we collected 2 g soil, which were frozen in liquid N_2_ and immediately stored at -80 °C for molecular analysis.

### DNA extraction, PCR amplification and Illumina sequencing

Prior to DNA extraction, the frozen BK, RS and RTS samples were ground and homogenized in a 2010 Geno/Grinder at 1000 rpm for 5 × 30 s. We extracted DNA from 0.25 g of each BK, RS and RTS sample using a DNeasy PowerLyzer PowerSoil Kit (QIAGEN) following the manufacturer’s protocol. DNA concentrations were measured with Qubit dsDNA HS (High Sensitivity) Assay Kit (Invitrogen, Thermo Fisher Scientific) and 50 µl were sent to Novogene for library preparation and sequencing. Briefly, DNA was diluted to 1 ng/µl using sterile water. The prokaryotic 16S rRNA gene V3-V4, the fungal ITS2 region, and the eukaryotic 18S rRNA gene V4 region, were amplified using specific primers 341 F/806R – 470 bp [27]; gITS7 / ITS4–380 bp [28]; and TAReuk454FWD1/TAReukREV3–417 bp [29], respectively, with the barcode. All PCR reactions were carried out with Phusion^®^ High-Fidelity PCR Master Mix (New England Biolabs). PCR products was mixed at equal density ratios. The mixed PCR products were purified with Qiagen Gel Extraction Kit (Qiagen, Germany). The libraries generated with NEBNext^®^ UltraTM DNA Library Prep Kit for Illumina and quantified via Qubit and Q-PCR, were finally sequenced on Novaseq6000 platform (Illumina). After sequencing, paired-end reads were assigned to samples based on their unique barcodes and truncated by cutting off the barcode and primer sequences.

### Sequence data and statistical analysis

Bacterial 16S rRNA, fungal ITS and eukaryotic 18S rRNA sequences were analyzed using the DADA2 (v. 1.12) [30] in the R statistical package (R Core Team, Vienna, Austria). Briefly, raw reads were quality filtered and trimmed (filterAndTrim - maxN = 0, maxEE = 2, truncQ = 2), followed by error learning (learnErrors), dereplication (derepFastq) and merging of forward and reverse reads (mergePairs) prior to the construction of the sequence table (makeSequenceTable) and chimera removal (removeBimeraDenovo). Taxonomy was assigned using the reference databases SILVA version 128 [31], the 2020 release of the UNITE database [32] and SILVA version 132 18S train set (https://benjjneb.github.io/dada2/training.html), for16S rRNA, ITS and 18S rRNA, respectively, using the implementation of the naive Bayesian classifier in dada2 R package. Unassigned ASVs at kingdom level or ASVs assigned as chloroplast or mitochondrial sequences were removed from all the datasets. ASVs assigned to fungi and Chloroplastida (mainly chloroplast and algae) sequences were removed from the 18S rRNA dataset. Statistical analyses and visualizations were carried out in R using vegan (v2.5.7) [33], phyloseq (v1.34.0.) [34], and ggplot2 (v3.3.2) [35] packages. Alpha diversity metrics observed richness and Shannon diversity were computed and statistically significant differences between groups estimated using Wilcoxon rank-sum test in the stat_compare_means function from ggpubr (0.4.0) (https://cran.r-project.org/web/packages/ggpubr/index.html). The ASV tables were transformed to relative abundances prior to beta diversity analysis. Bray-Curtis dissimilarity matrices were visualized, using unconstrained principal coordinates analysis (PCoA). Permutation analysis of variance (PERMANOVA) statistical tests were performed to determine the effects of experimental factors on the community dissimilarity using “adonis” in the vegan package, with 1000 permutations. Additionally, we performed Aitchison PCA to calculate beta diversity with feature loadings. This approach enables us to explore the taxonomic abundance changes responsible for sample clustering, and allow us to identity the specific taxa responsible for distinguishing between sample groups [36, 37].

Next, we performed differential abundance analysis between RKN-inoculated and non-inoculated rhizosphere and root samples using a Zero-inflated Gaussian approach with cumulative sum scaling (CSS) normalization in the package metagenomeSeq (V.1.26.3) [38]. We performed correlation analysis to identify microbial and eukaryotic taxa that correlated with *M. incognita*. For this, we followed a previously described correlation approach [39]. *M. incognita* ASV was correlated with bacterial, fungal and eukaryotic ASVs separately. We present ASVs that were present in at least 10 samples with Spearman’s rank correlations > 0.2 for positive correlations and <-0.2 for negative correlations, and *p* < 0.05. Correlations were visualized in heatmaps.

Microbial co-occurrence networks in bulk soil, rhizosphere soil and in roots of *M. incognita* inoculated and non-inoculated plants from the five time points were constructed as described previously [21]. Briefly, bacterial, fungal and eukaryotic datasets were pooled and normalized, using the trimmed mean of M values (TMM) method using the BioConductor package EdgeR [40]. Microbial networks were constructed using ASVs that were present in at least 40 samples with Spearman’s rank correlations > 0.6 for positive correlations and <-0.6 for negative correlations, and *p* < 0.001. The correlated ASVs were visualized in networks with ASVs set as nodes and correlations as edges. Network properties including transitivity or clustering coefficient (the probability that the adjacent nodes of a node are connected) and mean degree (the average number of edges across all nodes in a network), density (fraction of all possible edges actually realized), average path length (APL) (average number of steps which would be required to reach from one node to another in the network) and modularity (measures how well the network is organized into distinct modules) were computed, using the “igraph” package [41].

The robustness of each co-occurrence network was further tested by employing network attack tolerance strategies using the NetSwan package for R [https://cran.r-project.org/web/packages/NetSwan/index.html]. In this analysis, all networks were examined via systematic node removal using four strategies; (i) random removal, (ii) direct removal, where nodes were removed in descending order of their BNC (betweenness centrality) value (i.e., number of times a node is found on the shortest path between other nodes), (iii) targeted on nodes with the highest impact closeness (degree), and (iv) cascading removal, involving the recalculation of BNC values after each node was removed. Additionally, the impact on network connectivity loss was evaluated for each of these methods.

We further computed the top 5% of the ASVs having the most correlations (referred to as the keystone or hub taxa) in each of the constructed networks. Highly connected ASVs were identified and their relative abundances across time were examined.

## Results

***M. incognita*** infection reduces tomato growth parameters

Inoculation with *M. incognita* significantly reduced shoot height, number of branches, and dry and fresh weight at 60 dpi compared with the non-inoculated samples (Fig. 1B). The effect of *M. incognita* was not significant on tomato shoot parameters at earlier sampling times 0, 3, 7 and 30 dpi (Supplementary Table S1).

### Root microbiome composition

We obtained high quality reads of 8,848,383, 18,570,058, and 6,695,747 in the bacterial, fungal and eukaryotic datasets, respectively, from 180 samples (Supplementary Table S2). Because we profiled the fungal community separately using ITS specific primers, we removed fungal reads from the 18S dataset prior to the analysis, thus profiling the remaining eukaryotic community. The total number of sequence reads, rarefaction curves and read distributions visualizations are provided in Supplementary Figure S2. We observed time-dependent shifts in the relative abundances of specific microbial and eukaryote taxa in tomato compartments, with highly marked differences in relative abundances between RKN-inoculated and control samples at later developmental stages (Supplementary Figure S3). The relative abundance of Actinobacteria increased at 30 dpi but declined at 60 dpi in the non-inoculated samples (Supplementary Figure S3). The class Clostridia strongly decreased in RKN-inoculated samples compared to non-inoculated RTS at 60 dpi. Similarly, the relative abundance of Actinobacteria was higher in RKN-inoculated than in non-inoculated RTS at 60 dpi. The relative abundances of fungal genus *Fusarium* increased in roots at 30 dpi and 60 dpi in control, while *Plectosphaerella* was strongly enriched in RKN-inoculated RTS at 60 dpi (Supplementary Figure S3B). With increasing tomato development and time after RKN inoculation, Tylenchida (> 77% *M. incognita*) was strongly enriched in RKN-inoculated RTS samples at 30 dpi but declined at 60 dpi (Supplementary Figure S3C, Supplementary Table S3).

Alpha diversity (observed and Shannon diversity) generally revealed significant time dependent differences between RKN-inoculated and non-inoculated treatments in the different compartments (Supplementary Figure S4, S5). Generally, bacterial and fungal alpha diversity (observed and Shannon diversity) was lower in RTS than in BK and RS throughout the experiment. Bacterial and fungal observed richness and Shannon diversity were significantly higher in RKN-inoculated RTS at 30 dpi, but significantly lower compared with the non-inoculated RTS at 60 dpi (Supplementary Figure S4AB, S5AB). Fungal Shannon diversity was significantly different between RTS in RKN-inoculated and non-inoculated samples at 3 dpi, 30 dpi and 60 dpi (Supplementary Figure S5B). The bacterial Shannon diversity was significantly lower in RS of RKN-inoculated than non-inoculated samples at 3 dpi. In RS, bacterial observed and Shannon diversity and fungal Shannon diversity were significantly higher in RKN-inoculated than non-inoculated samples at 30 dpi. Observed fungal richness was significantly lower in RKN-inoculated RTS at 0 dpi and 3 dpi (Supplementary Figure S4B). A significantly higher fungal richness was further revealed in RKN-inoculated BK samples at 0 dpi and RS at 30 dpi. Eukaryotic alpha diversity (observed and Shannon diversity) was significantly higher in RS and RTS of non-inoculated than RKN-inoculated plants at 0 dpi and 3 dpi and only RTS at 30 dpi (Supplementary Figure S4C, S5C). However, eukaryote richness was higher in RS of RKN-inoculated samples at 30 dpi.

**Fig. 2:**
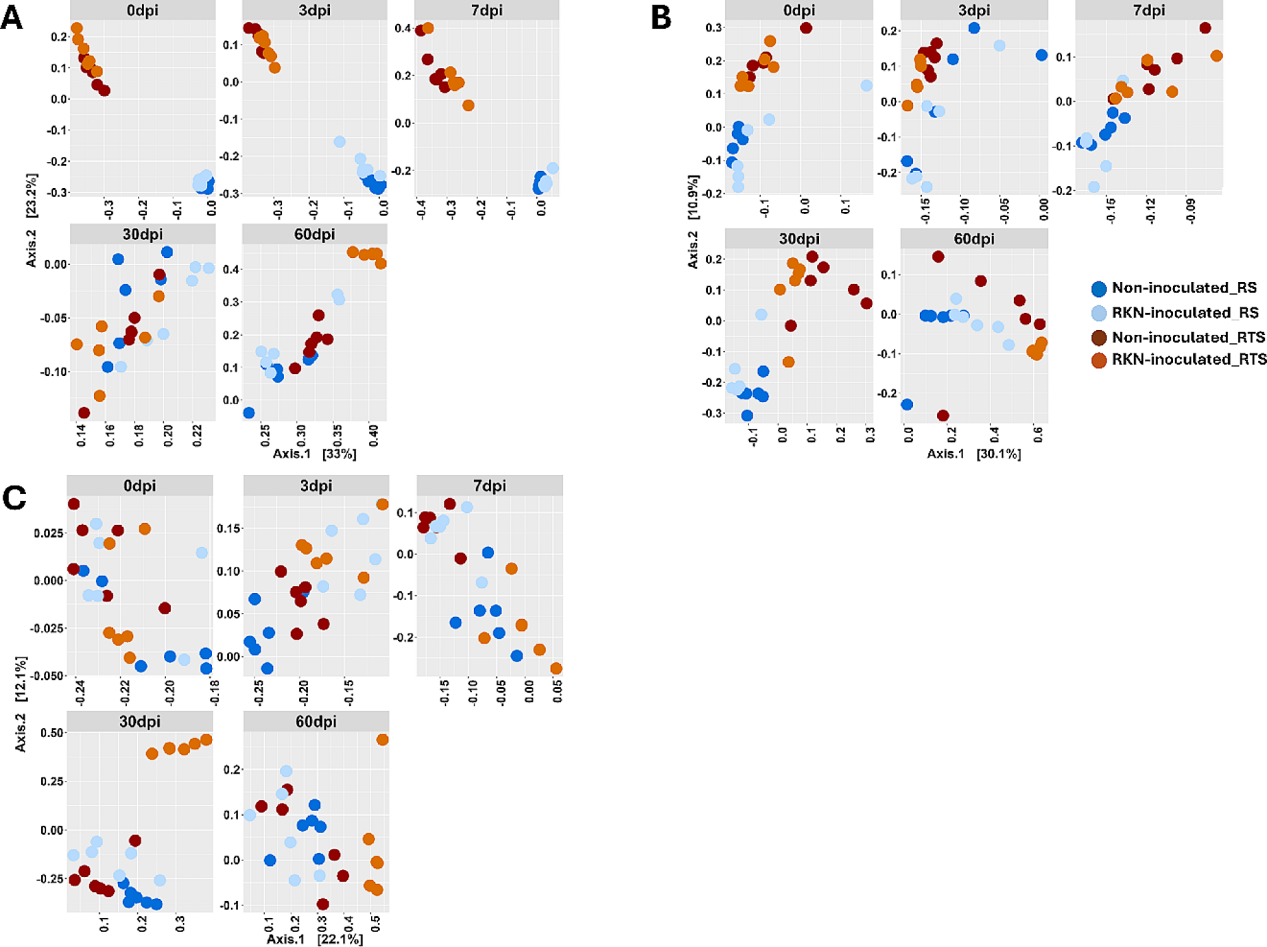
Principal coordinates analysis (PCoA) plots of **A**) bacterial **B**) fungal and **C**) eukaryotic communities in rhizosphere soil (RS) and in roots (RTS) of RKN inoculated and non-inoculated tomato plants 0–60 days post inoculation (dpi)

The composition of bacterial and fungal communities varied pronouncedly between the rhizosphere and root compartments at early timepoints. This difference diminished at the late sampling dates, where the effect of RKN inoculation manifested with more pronounced differences between the RKN-inoculated and non-inoculated treatment (Fig. 2A, B, Supplementary Figure S6A, B). In the eukaryotic dataset, differences in community composition between compartments and between treatments became clearer at later experimental stages, 30 dpi and 60 dpi (Fig. 2C, Supplementary Figure S6C). Generally, differences between inoculated and non-inoculated treatments were more pronounced in RTS than in RS. PERMANOVA analysis using rhizosphere and root datasets revealed that the plant compartment exerted the overall strongest effect on the bacterial and fungal community composition (bacteria; R^2^ = 0.17, fungi; R^2^ = 0.27, *p* < 0.001 for all communities; Table 1). For fungal communities, RKN inoculation had a small, yet significant effect on the community composition (R^2^ = 0.01, *p* < 0.05; Table 1). Seen over the entire experimental period, plant compartment and RKN inoculation had small, but significant effects on the eukaryotic communities (compartment; R^2^ = 0.04, inoculation; R^2^ = 0.03, *p* < 0.001; Table 1). A significant interaction between compartment and RKN inoculation (R^2^ = 0.02, *p* < 0.01; Table 1) was also detected in the eukaryotic community. These significant variations were similarly observed when using datasets including BS (Supplementary Figure S6). PERMANOVA for the individual sampling times revealed that the composition of bacterial and fungal communities varied considerably between RS and RTS compartments from the very early sampling dates (0–7 dpi) (0.67 ≤ R^2^ ≥ 0.73 for bacteria, and 0.20 ≤ R^2^ ≥ 0.27 for fungi; *p* < 0.001), whereas RKN inoculation had none to very limited impact on bacterial and fungal communities at this early stage of the experiment (Table 1, Supplementary Table S4). However, at the two late sampling dates, the effect of RKN-inoculation on bacterial and fungal communities was pronounced (Table 1). For the eukaryotic community, the pattern was different, as RKN inoculation affected their composition from the beginning of the experiment to the end (Table 1). For bacteria, fungi and eukaryotes, the interaction between compartment and RKN-inoculation became stronger with time, as the effect of RKN inoculation was more pronounced in the root compartment than in the rhizosphere at the late sampling times. A further pairwise comparison confirmed significant effects of RKN-inoculation at different sampling times (Supplementary Table S5).

**Table 1:** Summary of permutational analysis of variance (PERMANOVA) using the “adonis” test on Bray-Curtis distance matrices for bacterial, fungal and eukaryotic community dissimilarity assessment using 1,000 permutations. Datasets without bulk soil (BK) was used for this analysis

Dataset	Factors	Bacteria (16 S) *R*^2^	Fungi (ITS) *R*^2^	Eukaryotes (18 S) *R*^2^
Whole	Sample type	0.17***	0.27***	0.04***
	treatment	ns	0.01*	0.03***
	Sample type x treatment	ns	ns	0.02**
0 dpi	Sample type	0.71***	0.20***	0.06*
	treatment	ns	0.08*	0.23***
	Sample type x treatment	ns	ns	0.08**
3 dpi	Sample type	0.67***	0.27***	0.07*
	treatment	0.04*	0.08*	0.22***
	Sample type x treatment	ns	ns	0.08**
7 dpi	Sample type	0.73***	0.27***	ns
	treatment	ns	0.06*	0.09**
	Sample type x treatment	ns	ns	0.21***
30 dpi	Sample type	0.20***	0.33***	0.19 ***
	treatment	0.18***	0.09**	0.26***
	Sample type x treatment	ns	0.07*	0.16***
60 dpi	Sample type	0.23***	0.28***	0.15 ***
	treatment	0.28***	0.20***	0.13***
	Sample type x treatment	0.12***	0.11**	0.11**

Significance of test indicated as ***, *p* < 0.001; **, *p* < 0.01; *, *p* < 0.05. The ns denotes not statistically significant and R^2^ is the proportion of variation explained

PCA plot with taxa loading further revealed bacteria, fungal and eukaryote ASVs driving community composition differences between the RKN-inoculated and non-inoculated RS and RTS samples at the different sampling times (Supplementary Figure S7). Bacterial taxa including *Salinarimonas*,* Acidaminobacter* and *Fusibacter* were associated with inoculated RS samples at 30 and 60 dpi, while *Romboustia* and *Nesterenkonia* were highly associated with RKN-inoculated RTS samples at 60 dpi (Supplementary Figure S7A, B). The genus *Youngiibacter* was highly abundant in the non-inoculated RS and RTS samples at 60 dpi. Fungal PCA plots revealed that *Plectosphaerella* abundance caused late-stage fungal community shifts in RKN-inoculated RTS at 60 dpi (Supplementary Figure S7C, D). The fungal family Sebacinaceae strongly defined the RKN-inoculated RTS samples at 30 dpi. In the eukaryotic dataset, uncultured *Jakobida* and Diplogasterida were strongly associated with non-inoculated and RKN-inoculated RS samples, respectively at 60 dpi (Supplementary Figure S7F). Similarly, both uncultured *Jakobida* and Tylenchida defined the RKN-inoculated RTS samples at 60 dpi.

### Differential analysis

**Fig. 3:**
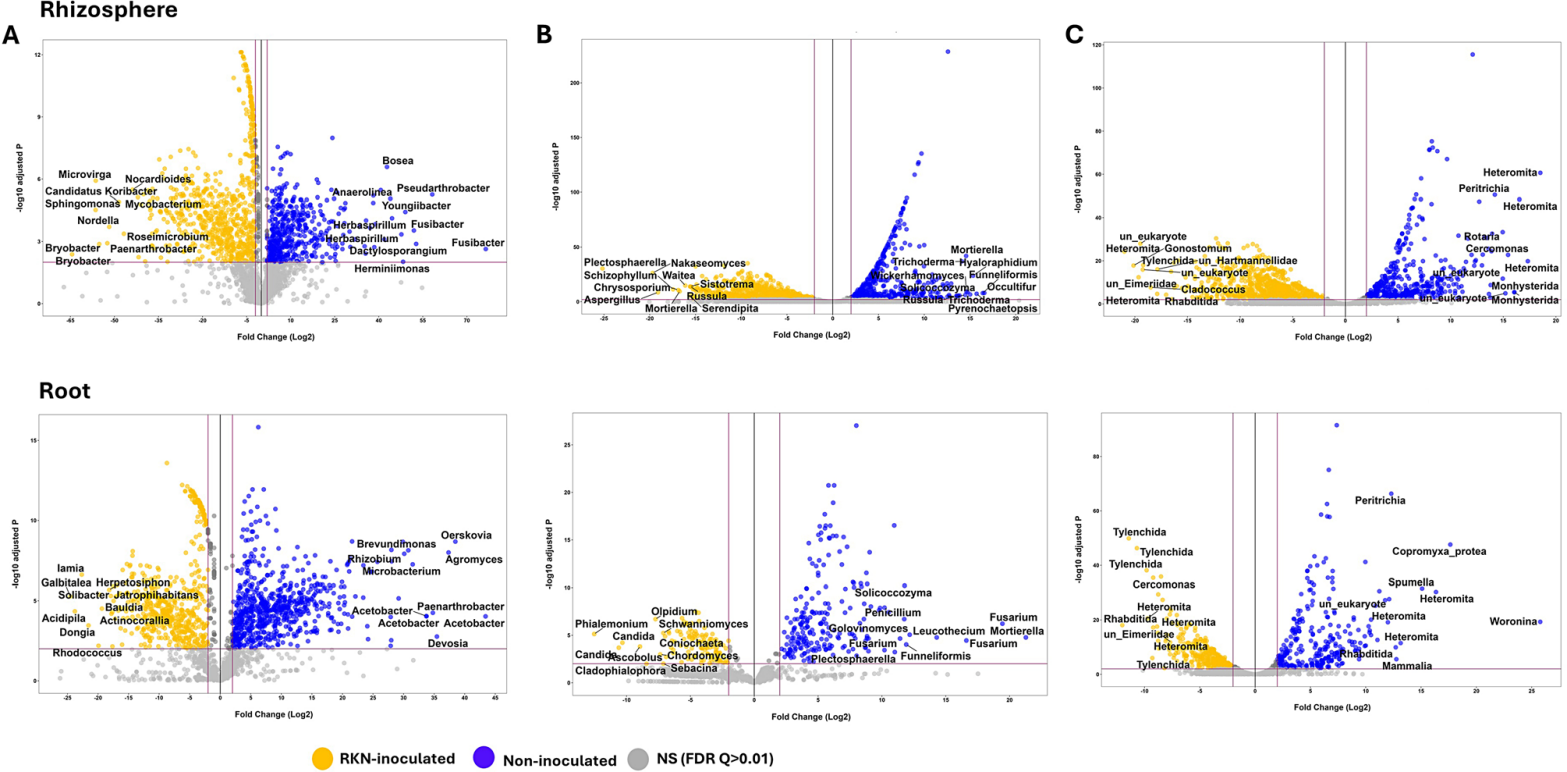
Volcano plot visualization of differentially abundant **A**) bacterial **B**) fungal and **C**) eukaryotic taxa (at ASV level) between RKN-inoculated and non-inoculated tomato rhizosphere and roots at different days post inoculation (dpi). Each point represents an individual ASV assigned to corresponding taxon. The position along the x-axis represents the direction of fold change. The red line shows the threshold of significantly differential ASVs (|log2(FC)|>=2). Taxa names of the 10 most differentially abundant taxa are shown in each sub-figure. Differentially enriched ASVs in non-inoculated and RKN-inoculated samples are shown in blue and yellow dots, respectively

We further explored variation in community composition by identifying ASVs whose abundances were differentially affected in response to *M. incognita* inoculation in the plant compartments and at different sampling times. We found many taxa in the RS and RTS with different abundances in RKN-inoculated and non-inoculated treatments (Fig. 3, Supplementary Table S6). For instance, bacterial taxa *Mycobacterium*, *Bryobacter*, *Sphingomonas*,* Bdellovibrio*, and *Bauldia* were enriched in RS and RTS of RKN-inoculated samples, whereas *Brevundimonas* was enriched in both compartments of non-inoculated plants (Fig. 3A, Supplementary Table S6). *Fusibacter*, *Herbaspirillum*, *Youngibacter* and *Pseudarthrobacter* were strongly enriched in RS of non-inoculated and similarly for *Acetobacter*, *Devosia* and *Oerskovia* in RTS of non-inoculated samples. Also, a broad range of fungal taxa, e.g. *Plectosphaerella*,* Aspergillus* and *Nakaseomyces* were enriched in RS of RKN-inoculated plants, while *Occultifur* and *Funneliformis mossae* increased in RS of non-inoculated. The fungal taxa *Phialemonium inflatum*,* Exophiala* and *Candida subhashii* were enriched in RTS of RKN-inoculated plants while *Fusarium solani* and *Mortierella elongata* were significantly more abundant in RTS of non-inoculated samples (Fig. 3B, Supplementary Table S6). For eukaryotes, we saw increased abundances of taxa including e.g. Rhabditida, *Gonostomum*, *Cladococcus* and uncultured Eimeriidae in RKN-inoculated RS, while e.g. *Cercomonas*, Cercozoa, Monhysterida and Peritrichia were enriched in the non-inoculated rhizosphere samples (Fig. 3C, Supplementary Table S6). Similarly, in roots, the order Tylenchida (mainly *M. incognita*) was strongly enriched in RKN-inoculated samples while Peritrichia, *Woronina* and *Copromyxa protea* increased in the non-inoculated samples.

Further differential analysis revealed additional differentially abundant taxa in RS and RTS of RKN-inoculated and non-inoculated plants at individual timepoints (Supplementary Figure S8). The abundance of bacterial genus *Sphingomonas* significantly increased in RS of RKN-inoculated setups while *Mycobacterium* and *Norcardioides* were consistently enriched in RTS of RKN-inoculated plants at each individual sampling time (Supplementary Figure S8A, B). In RTS of inoculated plants, we found an initial increase in the abundance of *Mesorhizobium*, but this taxon declined, and *Rhizobium*, *Solibacter*, *Azospirillum* and *Pelagibacterium* increased at later stages in inoculated RTS. Fungal genus *Mortierella* was enriched in RS of RKN-inoculated plants at each individual sampling time, but only at 7 and 30 dpi in RTS of RKN-inoculated plants (Supplementary Figure S9A, B). *Solicoccozyma* and *Trichoderma* were also enriched in RTS of RKN-inoculated plants at 3 and 7 dpi. We did not identify differentially abundant fungal taxa when comparing RTS of RKN-inoculated and non-inoculated setups at 60 dpi. Although ASVs assigned as *Heteromita* and uncultured Eimeriidae were differentially distributed between the RKN-inoculated and non-inoculated RS and RTS samples, specific ASVs belonging to these taxa were enriched or depleted in a time-dependent manner (Supplementary Figure S10A, B). Protist taxa such as the genus *Cercomonas* and uncultured Eimeriidae were differentially abundant in RS of RKN-inoculated setups compared with the non-inoculated samples at each individual sampling time. The order Tylenchida (mainly *M. incognita*) was significantly enriched in RTS of RKN-inoculated plants at 30 and 60 dpi (Supplementary Figure S10C).

### Correlation between ***M. incognita*** and bacterial, fungal and eukaryotic taxa

**Fig. 4:**
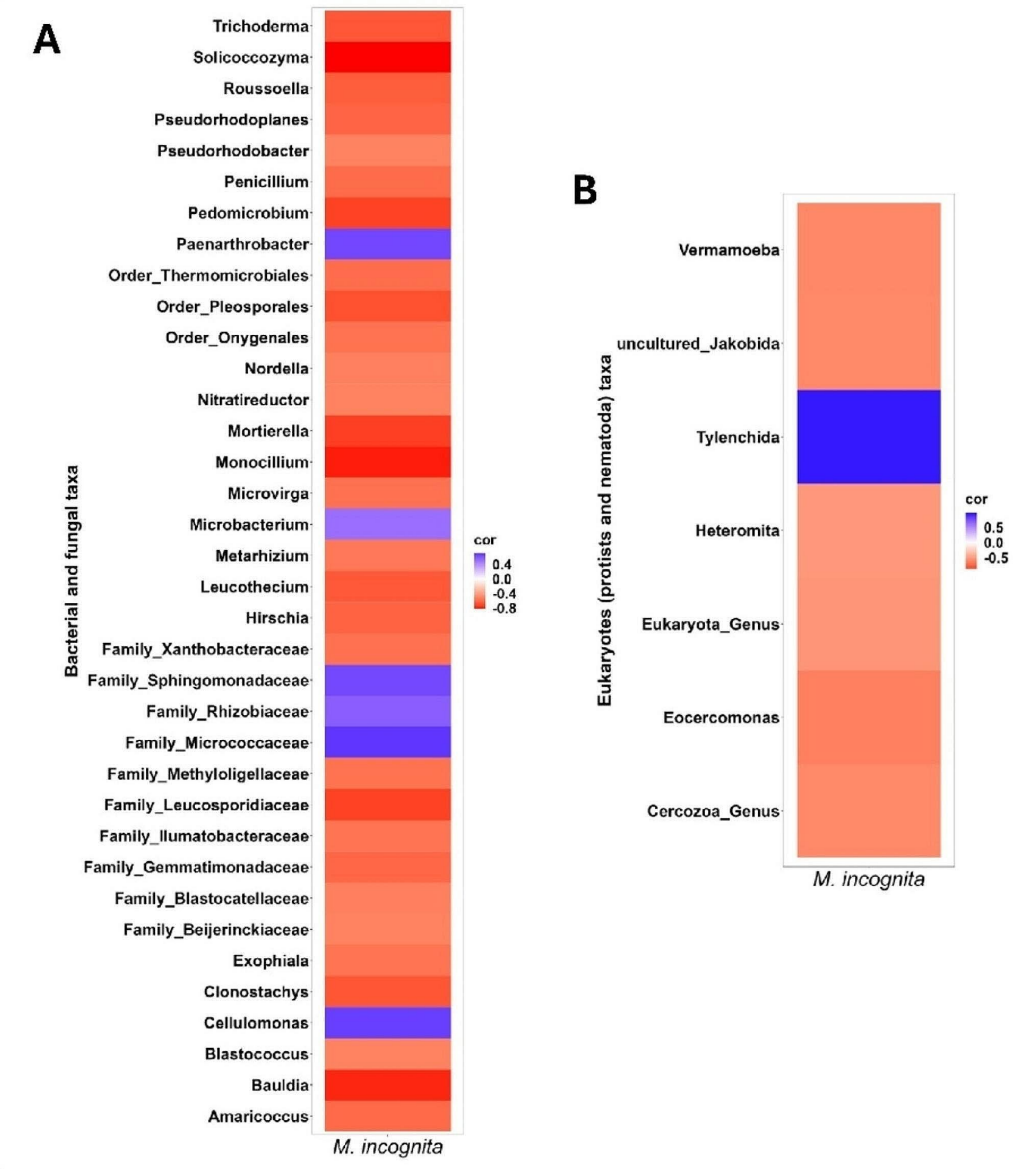
Heat map showing significant correlations between RKN (*M. incognita*) and bacterial and fungal taxa and **B**) eukaryotic taxa. Bacterial, fungal and eukaryotic data from all time points was pooled and used for the analysis. ASVs that were present in at least 10 samples with Spearman’s rank correlations > 0.2 for positive correlations and <-0.2 for negative correlations, and *p* < 0.05 were used. Blue color indicates positive correlations and red color indicates negative correlations

We performed correlation analysis to identify microbial taxa potentially associating with *M. incognita*. In most cases, correlations were negative (Fig. 4). Bacterial genera including *Pedomicrobium*, *Bauldia*, *Hirschia*, *Pseudorhodoplanes*, *Pseudorhodobacter* and families Methyloligellaceae, Gemmatimonadaceae and Xanthobacteriaceae correlated negatively with *M. incognita* (Fig. 4A). A few bacterial taxa such as the genera *Paenarthrobacter*, *Cellulomonas*, the families Micrococcaceae, Rhizobiaceae, Sphingomonadaceae correlated positively with *M. incognita.* Fungal taxa including *Monocillium*, *Solicoccozyma*, *Trichoderma*, *Mortierella*, and *Clonostachys* correlated negatively with *M. incognita.* A few protist taxa including the phylum Cercozoa, the genera *Eocercomonas* and *Vermoamoeba* correlated negatively with *M. incognita*, but generally correlations between *M. incognita* and eukaryotes were weaker than correlations with bacterial and fungal taxa (Fig. 4B).

### Co-occurrence network analysis

**Fig. 5:**
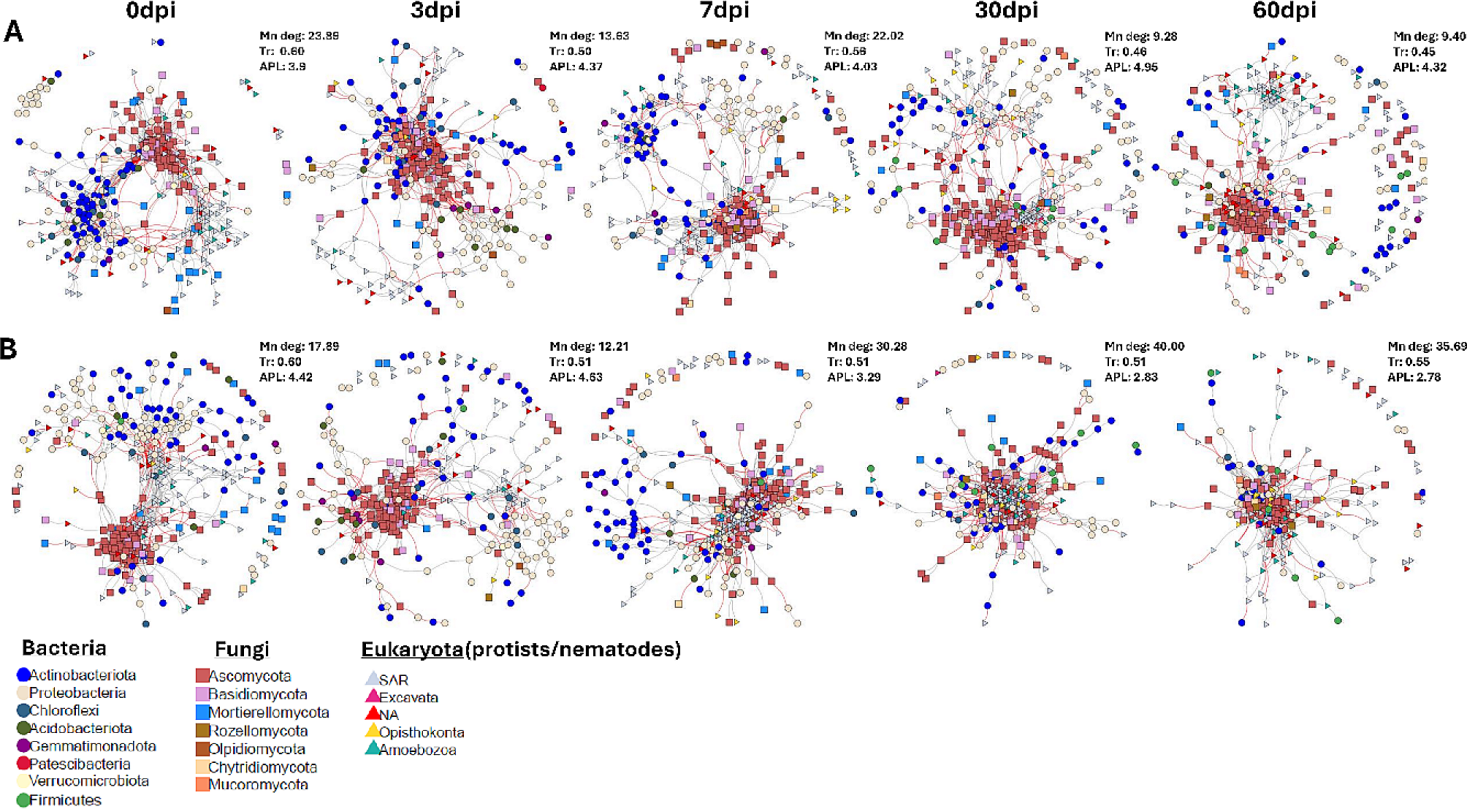
Microbial-eukaryotic co-occurrence networks in (A) non-inoculated and (B) RKN-inoculated tomato roots at 0–60 days post inoculation (dpi). The grey and red edges represent positive and negative correlations, respectively

We performed co-occurrence network analyses to examine the community dynamics between bacteria, fungi and eukaryotes using pooled RS and RTS datasets. The network properties are provided in Supplementary Table S7. Generally, the number of nodes and edges (both positive and negative) differed between inoculated and non-inoculated networks. The highest number of positive edges (inoculated: 5462 and non-inoculated: 4061), and negative edges (inoculated: 872 and non-inoculated: 1433) were found among bacteria. The lowest number of positive and negative correlations were observed between fungi and eukaryotes (inoculated: 13 and non-inoculated: 30), and within eukaryotes (inoculated: 2 and non-inoculated: 24) (Supplementary Table S7). Network structures of RKN inoculated and non-inoculated plants changed along different trajectories during the course of the experiment (Fig. 5A, B). Overall, in the non-inoculated plants, we saw a development from densely connected networks with many edges and high density at the early sampling times towards more loosely structured networks with weaker network topologies at later growth stages (Fig. 5A). In contrast to this development, networks of inoculated plants became more densely connected with time (Fig. 5B). Network metric such as mean degree decreased from 14 to 24 at day 0–7 dpi to 9 at 30–60 dpi in the non-inoculated plants, whereas in RKN-inoculated plants mean degree increased from 12 to 18 at 0–3 dpi to 30–40 at 7–60 dpi. The average path length was shortest, while transitivity and density were higher in the RKN-inoculated networks compared with the non-inoculated networks, specifically at 30 and 60 dpi (Fig. 5A**B**, Supplementary Table S7). Similarly, we observed that modularity increased from 0.29 to 0.38 at 0 dpi and 3 dpi, respectively, but decreased at later stages, 7, 30 and 60 dpi in the RKN-inoculated networks. In contrast, in the non-inoculated networks, modularity was initially stable but peaked at 7, 30 and 60 dpi.

**Fig. 6:**
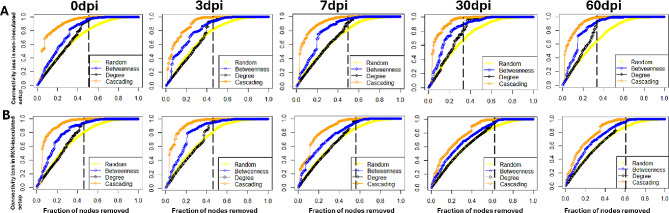
Tolerance to attack of **A**) non-inoculated and **B**) RKN-inoculated networks, at 0–60 days post inoculation (dpi) using change in connectivity as a function of the fraction of removed nodes. Dashed lines show the maximum fraction of nodes to be removed for 100% connectivity loss (highest breakdown of network). The four sustained attack strategies to test tolerance included random removal, direct removal of nodes with highest betweenness centrality, target on nodes with the highest impact closeness (degree), and a combination of random and targeted on betweenness (cascading)

To test the robustness of RKN-inoculated and non-inoculated networks, we examined their tolerance to four node sustained attack strategies, i.e. random removal, direct removal of nodes with highest betweenness centrality, targeted on nodes with the highest impact closeness (degree), and a combination of random and targeted on betweenness (cascading). For all networks, random removal exhibited the least connectivity loss, whereas cascading caused the lowest tolerance. RKN-inoculated and non-inoculated networks at 0 and 3 dpi had similar robustness, as similar fraction of node removal was required to reach 90–100% loss in connectivity (cascading) (Fig. 6). At 7 dpi, differences in tolerance between non-inoculated and RKN-inoculated networks became apparent. At 30 and 60 dpi, removal of 35% nodes resulted in a 90–100% breakdown of non-inoculated networks, whereas similar disintegration of the RKN-inoculated network required at least 60% node removal. Together, these results suggest that RKN-inoculated networks were more robust than the non-inoculated networks (Fig. 6).

**Fig. 7:**
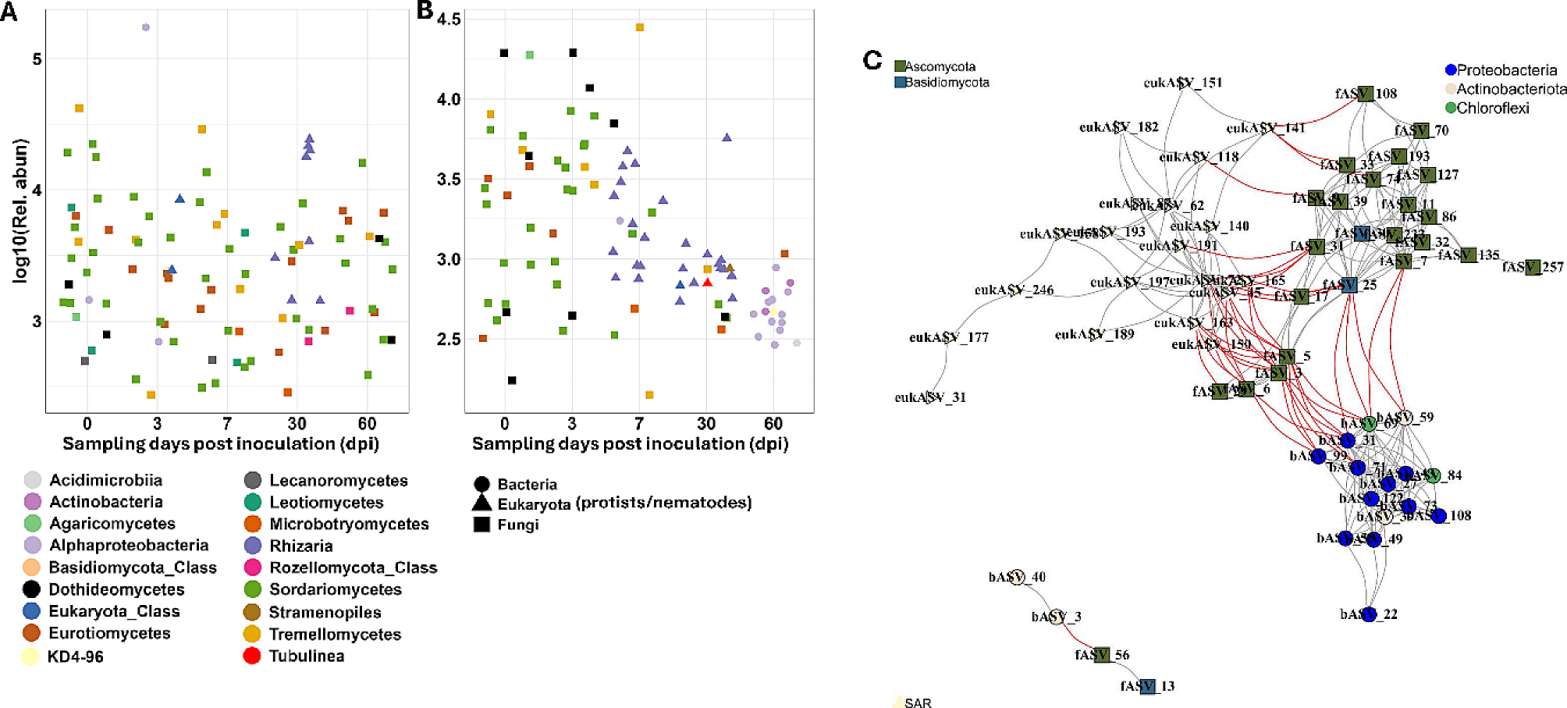
Hub taxa distribution and interactions. Relative abundance of hub taxa in **A**) non-inoculated and **B**) RKN-inoculated networks at 0–60 days post inoculation (dpi). Hub taxa was defined as the top 5% of ASVs with the most correlations in the network. **C**) Co-occurrence network of hub taxa revealing strong interkingdom negative correlations and intra-kingdom positive correlations. The grey and red edges represent positive and negative correlations, respectively

Moreover, in the RKN-inoculated networks we found a clear succession of hub taxa, where fungi dominated at the beginning of the experiment (0 and 3 dpi), whereas eukaryotes became the dominant hub taxa at 7 and 30 dpi, and finally, bacteria dominated at 60 dpi. In contrast, there was no clear temporal change in hub taxa dominance in the non-inoculated plants, where fungal hub taxa were predominant during the entire experimental period. In addition, the number of hub ASVs were relatively similar at the different sampling times, but decreased at 60 dpi in the RKN-inoculated network (Fig. 7A, B; Supplementary Table S7). We found that fungal and bacterial hubASVs belonged to the dominant plant associated taxa Sordariomycetes and Alphaproteobacteria and Actinobacteria, respectively. The majority of the eukaryotic hubASVs were assigned to Rhizaria. Further analysis of the hub members shows strong negative interkingdom correlations between bacterial, fungal and eukaryotic hub ASVs, while positive correlations dominate interactions between taxa from the same kingdom (Fig. 7C). Fungal hubASVs including fASV31 (order Onygenales) correlated negatively with few eukaryotic taxa including eukASV191 (*Heteromita*), eukASV45 (Thecofilosa) and eukASV72 (Glissomonadida). Similarly, fASV25 (*Solicoccozyma*), fASV3 and fASV5 (*Plectosphaerella*) and fASV6 (*Fusarium*) had antagonistic association with hubASVs of both bacteria and eukaryotes (Fig. 7C).

## Discussion

It is accepted that host associated microbiomes affect plant pathogens and parasites, either directly or indirectly, and thus ultimately influence interactions between plants and their pathogens and parasites [2, 5, 42]. Many investigations of the impact of the microbiota on plant parasitism focus on the bacterial and fungal communities, inadvertently overlooking the significance of microeukaryotes, although microbial feeding eukaryotes, notably protists and nematodes, are important for the activity and taxonomic composition of microbial communities [43]. The present study provides a comprehensive analysis by tracking the dynamics of bacterial, fungal and eukaryotic communities associated with tomato plants inoculated with RKN *M. incognita* and non-inoculated plants for 60 days. The experimental duration covers the entire life cycle of *M. incognita*, enabling a time-dependent profiling of communities structuring, and further exploring the dynamics of root microbiome interactions during *M. incognita* infection of tomato plants.

Our initial analysis confirmed the negative impact of *M. incognita* infection on plant performance with significant reductions in height, number of branches, and dry and fresh weight of RKN-inoculated compared to non-inoculated plants. Tylenchida reads (highly dominated by *M. incognita*) dominated the eukaryote community of RKN inoculated roots at 30 dpi, reflecting the progressed infestation of the roots. We note that the relative abundance of Tylenchida (and thus RKN) declined from 30 to 60 dpi. This probably reflects that RKN offspring developing in eggs contributed to the Tylenchida reads at 30 dpi, whereas a significant proportion of the offspring had hatched and migrated into the soil matrix at 60 dpi.

### RKN invasion altered microbial and eukaryotic communities.

The host associated microbiome is dynamic and both the host traits, external perturbations and intrinsic interactions drive community assembly and development through time [7, 21]. Our data revealed distinct abundances of specific microbial taxa in BK, RS and RTS of *M. incognita*-treated and non-inoculated tomato plants, thus confirming compartment specific enrichment in plants [44]. The high prevalence of bacterial taxa Alphaproteobacteria and Actinobacteria and fungal class Sordariomycetes has been previously reported [44–46]. These taxa are known to be predominant in soil and constitute core members of the plant microbiome involved in varying ecological roles that influence plant health [47, 48]. The bacterivorous flagellate *Heteromita* dominating the protist community, and increasing in RTS at later sampling times, is generally dominant in soils [49].

While microbial communities in soils have been reported to affect RKN invasion [5, 42, 50], root infections by pathogens or parasites result in overarching host configurations that overall affect host associated microbial communities [21, 51, 52]. Our results show that the relative abundances of specific microbial and eukaryote taxa were markedly different in the RKN-inoculated and non-inoculated RS and RTS of tomato plants, especially after the infection had time to develop, at 60 dpi. For instance, bacterial taxa Actinobacteria and fungal genus *Plectosphaerella* were strongly enriched in RKN-inoculated RTS at 60 dpi. Previous studies have reported enrichment of Actinobacteria in the rhizosphere of nematode-parasitized plants [51]. It has been reported that *Plectosphaerella* attaches to the surface of the J2s of *M. incognita* [4]. Further, *Plectosphaerella* was reported in rhizosphere soils of *Luffa cylindrica* plant infected with *M. incognita* [53], underpinning an association between *M. incognita* root invasion and *Plectosphaerella*. *Plectosphaerella* spp. are pathogens on tomato [54], and the enhanced abundance of the genus in RKN inoculated roots could suggest that RKN root penetration facilitates *Plectosphaerella* infections, as is also the case for other fungal pathogens, e.g. *Fusarium oxysporum* and *Rhizoctonia solani* [55].

The reduced species richness and diversity in roots compared with bulk and rhizosphere supports previous studies and further corroborates the selective role of the rhizoplane in regulating microbial entry into the root [44, 56]. Moreover, the lower microbial and eukaryotic species richness and diversity in rhizospheres and roots of RKN inoculated than non-inoculated plants is consistent with earlier studies [2, 57]. While several factors including nematode parasitism, host genotype and developmental stage and management practices contribute significantly to variations in plant-associated rhizosphere and root bacterial and fungal communities [2, 7, 42, 58], our data revealed that *M. incognita* significantly alters both prokaryotic (bacteria) and microbial eukaryotic (fungal, protist and nematode) community composition and diversity. To our knowledge, this is among the first studies to examine the temporal dynamics of bacterial, fungal and eukaryotic communities during plant parasitic nematode infection of tomato.

For bacterial and fungal communities, the most noticeable distinctions between RKN-inoculated and non-inoculated roots became evident at 60 dpi. RKN inoculation had a progressively increasing impact on microbial and eukaryotic community structures during the duration of the experiment, while the distinction between rhizosphere and root communities became less apparent with time. These observations were also consistent with the clustering of distinct taxa on the unconstrained PCA results.

Community changes during RKN-parasitism could be explained by the movement of J2s from soil into root tissues, followed by enhanced parasitic activities, reproduction and interactions with microbes. RKN parasitism is accompanied by plant secretion of an array of antioxidants, such as peroxidases, peroxiredoxin, thioredoxins, glutathione-s-transferase [10, 59]. Besides these plant responses, changes in community structures may be linked to defense responses resulting from the complex immune system of plants. This may involve the overall production of phytoalexins, such as glycoalkaloid tomatine and flavonoids, as well as the release of volatile compounds when plants are subjected to nematode attacks [10]. Phytoalexins including flavonoids have modulating effects on the host-associated microbiomes [58, 60].

### RKN infection affected specific microbial and protist taxa

We found distinct enrichments and depletions of individual taxa in the rhizosphere and roots of RKN-inoculated and non-inoculated samples at individual timepoints. Time-dependent enrichment of bacterial taxa such as *Mycobacterium*, *Bdellovibrio*, *Norcardioides*,* Rhizobium* and *Azospirillum*, fungal taxa including *Mortierella*, *Solicoccozyma* and *Trichoderma*, and eukaryotic taxa *Cercomonas* and uncultured Eimeriidae in the roots of RKN inoculated compared with the non-inoculated samples indicates that changes associated with RKN parasitism affect these taxa. Previous studies have reported the potential suppressive effect of microbial taxa *Mycobacterium*,* Norcardioides*,* Rhizobium*, and *Azospirillum*,* Mortierella* and *Trichoderma* against plant parasitic nematodes including *M. incognita* [60, 61]. The increasing enrichment of these putatively RKN-antagonistic microbial taxa in the RKN inoculated plants could suggest a recruitment response mediated by the host [62, 63]. Oppositely, previously we found a higher prevalence of *Bdellovibrio* associated with live than inactive RKN, which could suggest that *Bdellovibrio* protects infective J2 against other microbial antagonists [19]. Root infection caused by RKN may also lead to changes in the root structure, potentially promoting the proliferation of specific microbial species [51]. Moreover, differentially enriched taxa in the roots of RKN-inoculated samples could also reflect that some microbial taxa gain access to root tissues via cracks formed between epidermal and cortical cells after RKN penetration [64]. Further, some of these microbes, e.g. *Bdellovibrio* and *Norcardioides*, also attach to the nematodes, which could be a means of transportation into the invaded roots [42]. It is interesting that protist genus *Cercomonas* was also enriched in RKN inoculated plants, as *Cercomonas* spp. may contribute to the suppression of pests and pathogens in the rhizosphere. For instance, *Cercomonas* sp. positively correlated with biological control agent *Bacillus* that suppressed *Fusarium oxysporum* in the rhizosphere of banana [65]. There is also evidence that *Cercomonas* sp. attachment to nematodes is lethal to nematodes [66]. It would therefore be interesting to verify if *Cercomonas* spp. contribute to the regulation of RKN infectivity.

### Specific microorganisms correlating negatively with ***M. incognita*** could have antagonizing effects

Potential antagonists of RKN within the host associated microbiota are candidates for implementing effective biocontrol strategies. The high number of negative correlations between RKN and microbial taxa, notably the Alphaproteobacterial families Methyloligellaceae, Xanthobacteriaceae and fungal genera *Mortierella*, *Trichoderma* and *Clonostachys* could indicate that the tomato root microbiome harbours RKN-antagonistic taxa. Several microbial species within these taxa have suppressive effects towards plant parasitic nematodes through direct parasitism or the production of nematode suppressive compounds [13]. *Clonostachys* species are known to produce several nematicidal compounds including, leptosins, chetoracin A, chaetocin, and gliocladines [67], extracellular chitinases [68] and serine proteases [69]. Root colonization by *Trichoderma* species have been shown to impede all stages of nematode parasitism, and also interferes with the jasmonic acid-pathway to increase tomato defence against *M. incognita* [70]. The negative correlation between *Mortierella* is interesting and aligns with several reports that document that *Mortierella* spp. are antagonistic towards *Meloidogyne* spp. [71, 72]. Büttner et al. [73] also reported that *Mortierella verticillate* hosts a toxin-producing bacterial genus *Mycoavidus* that disrupts nematode attack. For Alphaproteobacteria genera, including *Bauldia*,* Hirschia*,* Pedomicrobium*, *Pseudorhodoplane* and the fungal genera *Solicoccozyma* and *Monocillium* that were strongly negatively correlated with RKN abundance, no previous studies found similar associations.

Some of the taxa that correlated negatively with RKN, including known RKN-antagonists *Clonostachys* and *Mortierella*, but also *Nordella*, *Microvirga* and *Bauldia* were enriched in the rhizosphere or roots of RKN-inoculated plants. This lends support to the idea that pest-antagonistic taxa are recruited as a response to pest invasion [63].

Some protists including bacterial feeding Cercozoa, *Eocercomonas* and *Vermoamoeba* correlated negatively with *M. incognita*, but the correlations were generally relatively weak (i.e. spearman *r*>-0.5). Only a few protist taxa such as the relatively large vampyrellid amoebae are known to be nematophagous [43, 74]. However, the small Cercozoan flagellate, *Cercomonas* sp., killed the bacterial feeding nematode *Caenorhabditis elegans* [66]. It therefore remains speculative if negative correlations between protist taxa and *M. incognita* reflect antagonist interactions. Indirectly, taxon-selective feeding of bacterial feeding protists [75] may modulate interactions between nematodes and bacteria, which could also partly explain correlations between bacterial feeding protists and *M. incognita*.

*M. incognita* correlated positively with a few bacterial taxa. Positive correlations could indicate that the microbial taxa in question protect nematodes against antagonists or facilitate their invasion of roots, as we and others have previously proposed [11, 76]. Interestingly, Sphingomonodaceae correlated positively with *M. incognita* in the present study aligning with reported positive associations between this bacterial family and high RKN infestation of tomato roots [77]. However, it still remains elusive whether positive correlations between RKN and microbial taxa reflect conducive relationships between microbes and nematodes, and it probably depends on the specific microbial taxa involved. For instance, we see a positive correlation between *M. incognita* and *Microbacterium*, a genus that encompasses species that attach to and antagonize nematodes [78].

### Tripartite networks alter and reveal dynamic interactions during ***M. incognita*** infection

Our time-series analysis revealed distinct patterns of microbe-eukaryote interactions in RKN-inoculated and non-inoculated rhizosphere/root samples of tomato at different time points. The changes in the number of positive and negative co-occurrences between the inoculated and non-inoculated treatments indicate that interactions vary between benign and invaded ecological systems [79–81], and further support evidence of microbiome shifts during RKN infection. Positive and negative co-occurrences are characteristic of cooperative and antagonistic interactions within the microbiota associated with the host plant, respectively [79].

Topological metrics such as mean degree, transitivity and APL are used to examine the characteristics of community co-occurrence patterns during external perturbations [24, 82–84]. Across time, the RKN-inoculated networks became increasingly tightly clustered compared with the non-inoculated networks. Correspondingly, the mean degree increased, and APL decreased across time in RKN inoculated networks, whereas these metrics developed oppositely in non-inoculated networks. These distinct topological changes indicate the development of a more resilient community structure attained via enhanced cooperative or antagonistic interactions in response to RKN parasitism. An increasing mean degree, transitivity and low APL commensurate a network that is highly clustered while low transitivity signifies networks with loosely connected clusters. Moreover, the shorter average path length and network density (the observed proportion of total possible edges) found in the RKN-inoculated network defines a compact microbial community structure [85, 86] likely facilitating increased microbial cooperation and communication [87] during RKN parasitism. While the increasing modularity across time in the non-inoculated network could indicate an ongoing development of subcommunities into modules (smaller clustered functional niches) [85, 87, 88], the decrease in modularity at 7, 30 and 60 dpi in the RKN-inoculated network were likely disrupted modules. It is thus possible that the RKN-inoculated network underwent a highly dynamic structuring by reconfiguring weaker modules into a more centralized core clustering with fewer and stronger modules to resist the invasion.

The outcome of the network resistance tests corroborates a higher robustness to disturbances in the RKN-inoculated networks than in the non-inoculated networks. While random attacks, i.e. removal of random nodes (ASVs), impacted both networks least, attack strategies using cascading and centrality removals caused the earliest total network collapse, and the non-inoculated networks collapsed earlier than the RKN-inoculated networks at later post inoculation stages. These results further support the importance of nodes (ASVs) with higher centralities, for example hub species in maintaining network integrity and resilience against external perturbations [89].

Hub taxa have many connections to other community members and are considered key players maintaining the network stability and structure [79, 82, 90, 91]. Hub species also drive key processes that affect plant health including disease prevention and nutrient uptake, thus their loss under perturbations such as invasion scenarios, could affect plant health [90, 91]. As a result of their importance, hub taxa have been suggested as the focal species that can be targeted for developing novel sustainable management options [91]. Co-occurrence network analysis of hub taxa revealed strong negative interkingdom correlations and positive intra-kingdom correlations. Fungal and bacterial hubs belonged to the dominant plant associated taxa Sordariomycetes and Alphaproteobacteria, respectively. The majority of the eukaryotic hubs were assigned to Rhizaria, which through their predation on bacteria affect the overall plant microbiota [92–94]. Studies have reported a high connectedness between bacterial, fungal and some cercozoan members belonging to the Rhizaria supergroup, highlighting the importance of these interactions in microbiome community assembly and stability [95]. In the RKN-inoculated network, we found a temporal succession of hub taxa, where fungal hub taxa were predominant at the earliest stage, followed by eukaryotes at 7–30 dpi, and eventually bacterial hub taxa dominated at 60 dpi. This pattern was distinctly different from the situation in non-inoculated networks, where most hub taxa were fungi throughout the experiment. This difference emphasizes that nematode parasitism may impose changes in root associated microbiomes with strong implications for the intrinsic regulation of microbiome interactions.

## Conclusion

In this study, we examined the dynamic changes of the microbial and eukaryotic communities of *M. incognita* inoculated and non-inoculated tomato plants. Over time, community composition and structuring developed differently in RKN-inoculated and the non-inoculated root microbiomes, and differences were strongest with increasing growth and infection time. We found several differentially affected bacterial, fungal and eukaryotic taxa between RKN-inoculated and non-inoculated rhizosphere and roots samples at individual timepoints. For example, taxa that have previously been associated with RKN as well as putatively RKN-antagonistic bacterial and fungal taxa were enriched in roots and rhizospheres of RKN-inoculated and non-inoculated samples. These findings suggest that *M. incognita* parasitism affects specific microbial taxa and further indicate that distinct RKN parasitic phases are characterised by changes in the tomato associated microbiome. In addition, correlation analysis revealed microbial taxa that were potentially antagonistic against *M. incognita*, several of which were also enriched in plants exposed to RKN. Moreover, topological parameters and robustness testing suggested stronger networking in the *M. incognita* infected rhizospheres/roots compared with the non-inoculated networks. Hub species that modulate the community network structures and determine overall stability were also found to assume successional dynamics, coupled with specific antagonistic interkingdom interactions in the RKN-inoculated networks. However, additional experiments are needed to assess the functional roles of these taxa in response to RKN parasitism. In summary, these findings provide comprehensive insights into the complex interaction between RKN and host-associated microbiomes, that will guide hypotheses on microbiome-mediated suppression of pathogens/pests, for example, whether core functional prokaryotic and eukaryotic traits promote inhibition of parasitic root-knot nematodes.

### Electronic supplementary material

Below is the link to the electronic supplementary material.

Supplementary Material 1

Supplementary Material 2

Supplementary Material 3

Supplementary Material 4

## Data Availability

The MiSeq paired end reads obtained from the bacterial 16S rRNA gene (V3-V4), fungal ITS2 and eukaryote 18S rRNA gene regions have been deposited in NCBI SRA repository, under BioProject ID PRJNA1077257 and Submission IDs: SUB14229577, SUB14254567 and SUB14254822, respectively. Data analysis in R were performed using the pipeline and scripts adapted from [39].

## Abbreviations

M. incognita: Meloidogyne incognita
BS: Bulk soil
RS: Rhizosphere soil
RTS: Roots
dpi: Days post inoculation
ITS2: Internal transcribed spacer 2
16S rRNA: 16S ribosomal RNA
ASV: Amplicon sequence variant
PERMANOVA: Permutational multivariate analysis of variance
NCBI: National Center for Biotechnology Information
SRA: Sequence Read Archive
QIIME: Quantitative Insights into Microbial Ecology
