# Supplementary material for: Distinct changes in tomato-associated multi-kingdom microbiomes during Meloidogyne incognita parasitism
Source: Environ Microbiome. 2024 Jul 27;19:53. doi: 10.1186/s40793-024-00597-y (PMC11282865; doi:10.1186/s40793-024-00597-y)
Supplement: Supplementary file 4 — Supplementary Material 4 [file 40793_2024_597_MOESM4_ESM.pdf]

Supplementary file

Distinct changes in tomato-associated multi-kingdom microbiomes during *Meloidogyne incognita* parasitism

Enoch Narh Kudjordjie[1], Susana S Santos[1], Olivera Topalovic[2], Mette Vestergård[1]

[1] Department of Agroecology, Faculty of Technical Sciences, Aarhus University, 4200 Slagelse, Denmark

[2]Department of Biology, Section of Terrestrial Ecology, University of Copenhagen, 2100 Copenhagen, Denmark

Correspondence: Mette Vestergård (mvestergard@agro.au.dk)

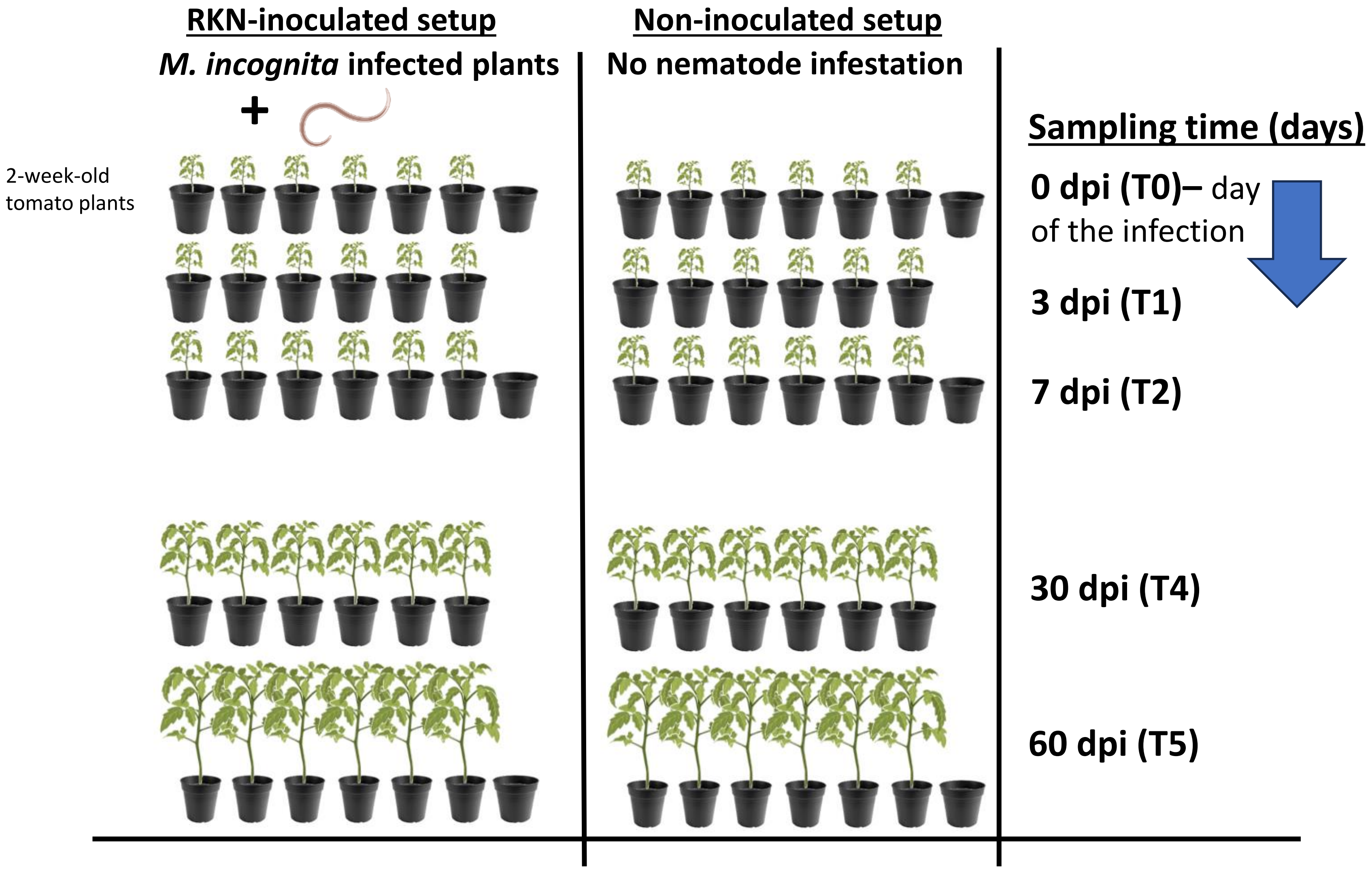

Figure S1: Experimental scheme used in this study. Two-weeks old tomato plants were inoculated with root-knot nematode (RKN) *M. incognita* (RKN-inoculated), while control setup had no RKN (Non-inoculated). Samples were taken at time 0 (day of inoculation) and subsequently at 3, 7, 30 and 60 days post RKN inoculation (dpi).

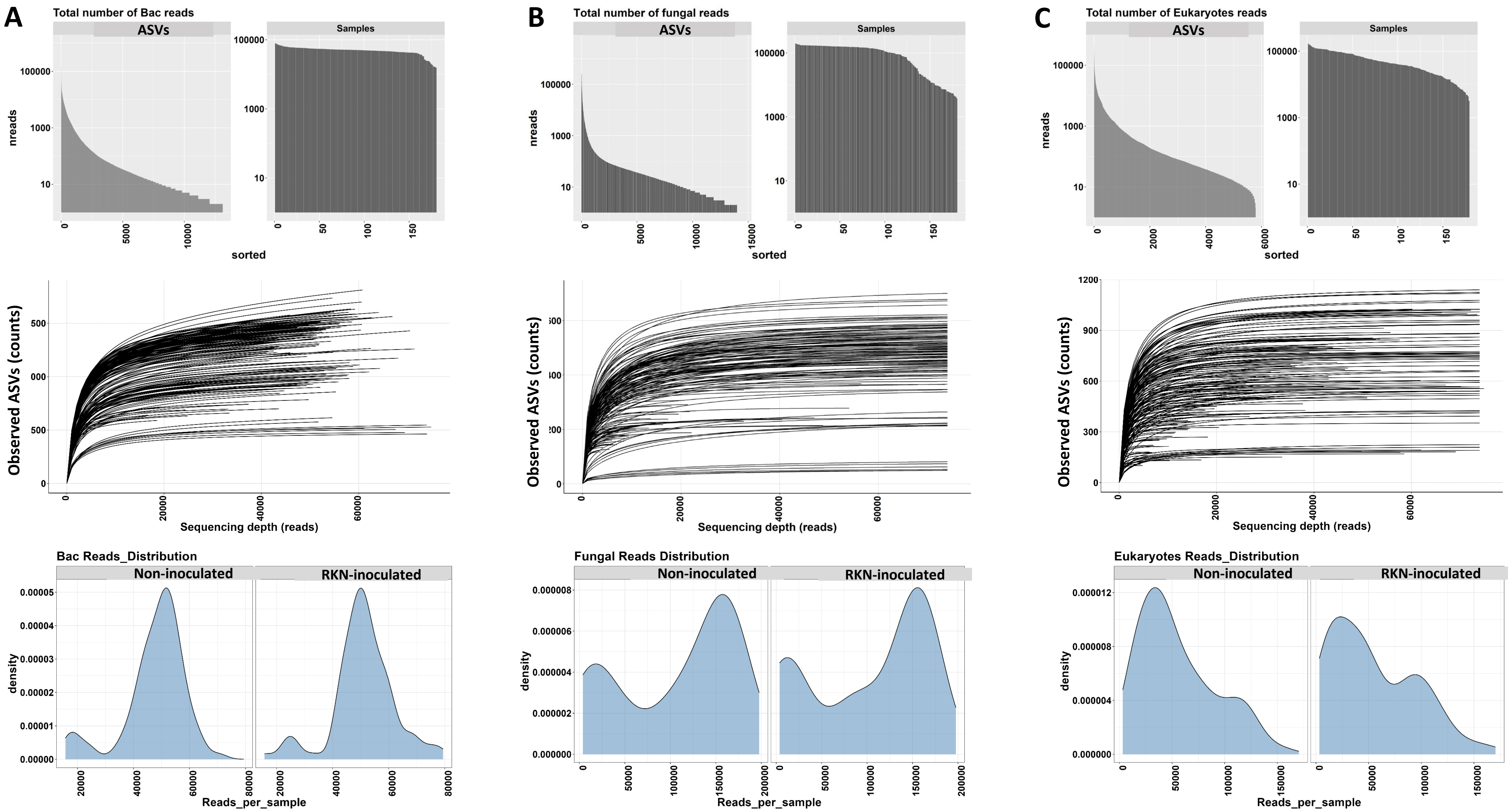

Figure S2: Distribution of (**A**) bacterial and (**B**) fungal and (**C**) eukaryotes sequence reads and ASVs, rarefaction curves showing the coverage of ASV richness (species richness in number of ASVs) as a function of sequencing depth (sample size in number of reads) and density plots.

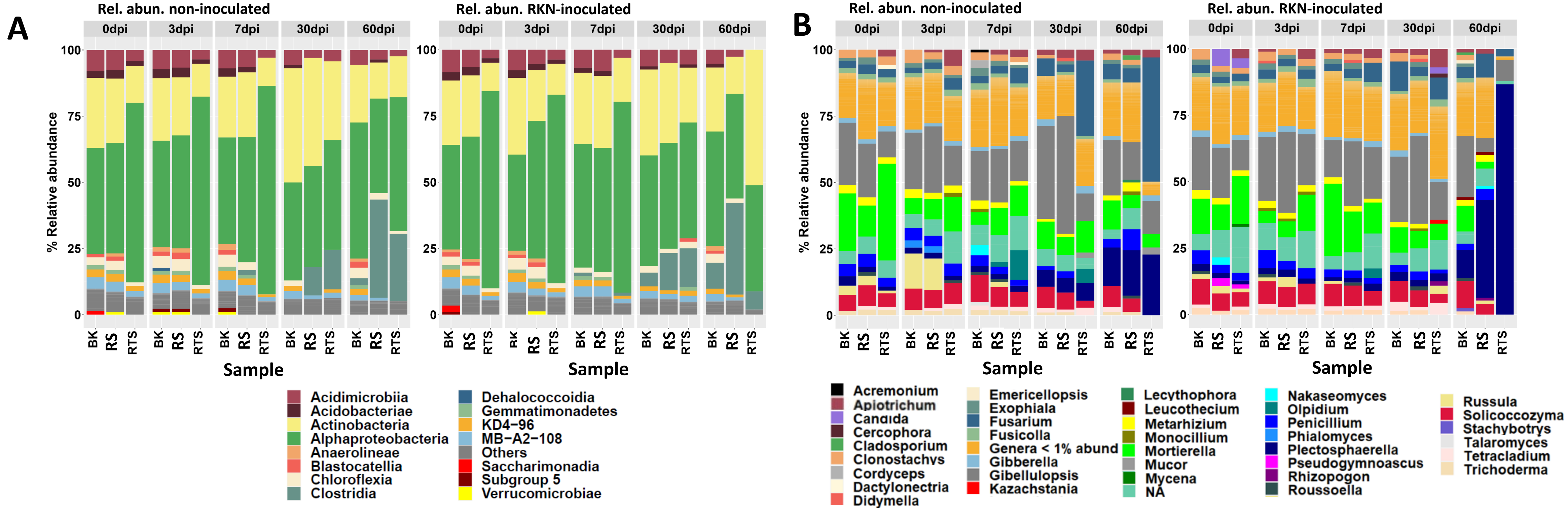

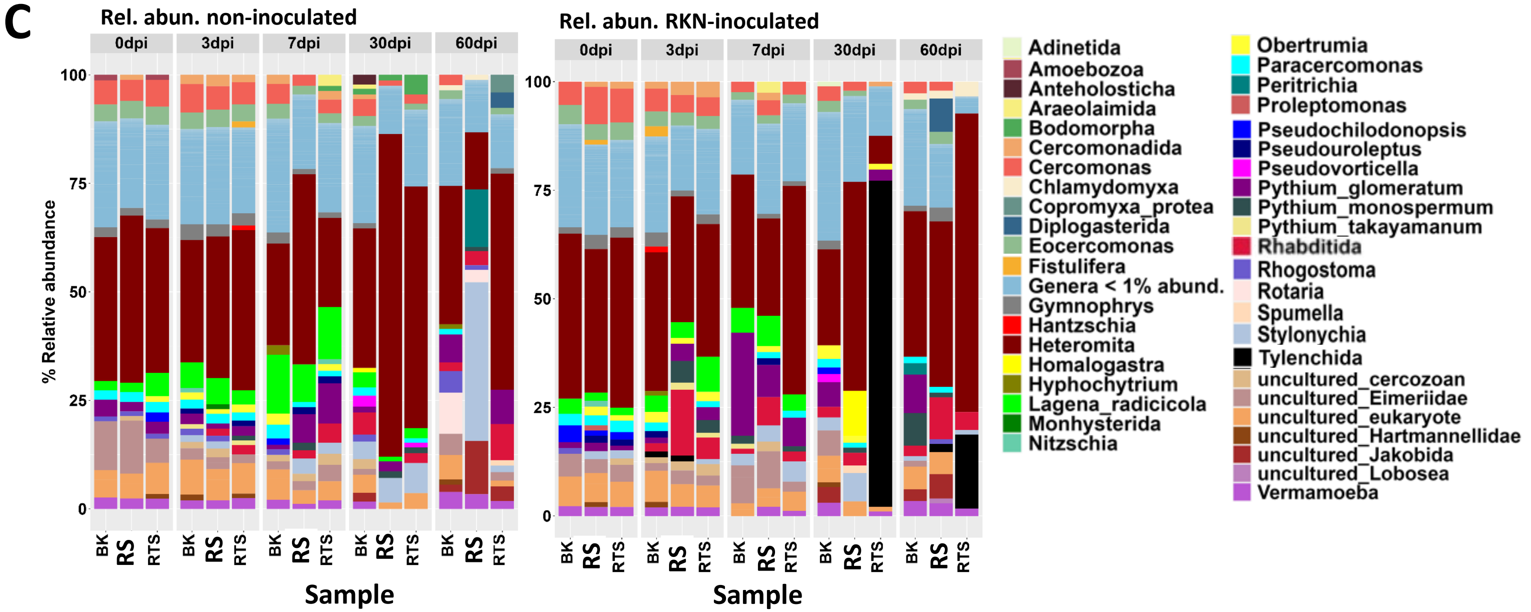

Figure S3. Relatives abundances of **A**) bacterial **B**) fungal and **C**) eukaryotes in the bulk soil (BK), rhizosphere (RAS) and roots (RTS) samples of RKN-inoculated and non-inoculated setups, at different sampling time points. Tylenchida had >77% *M. incognita* at 30 and 60 dpi (see Supplementary Table S3 for details).

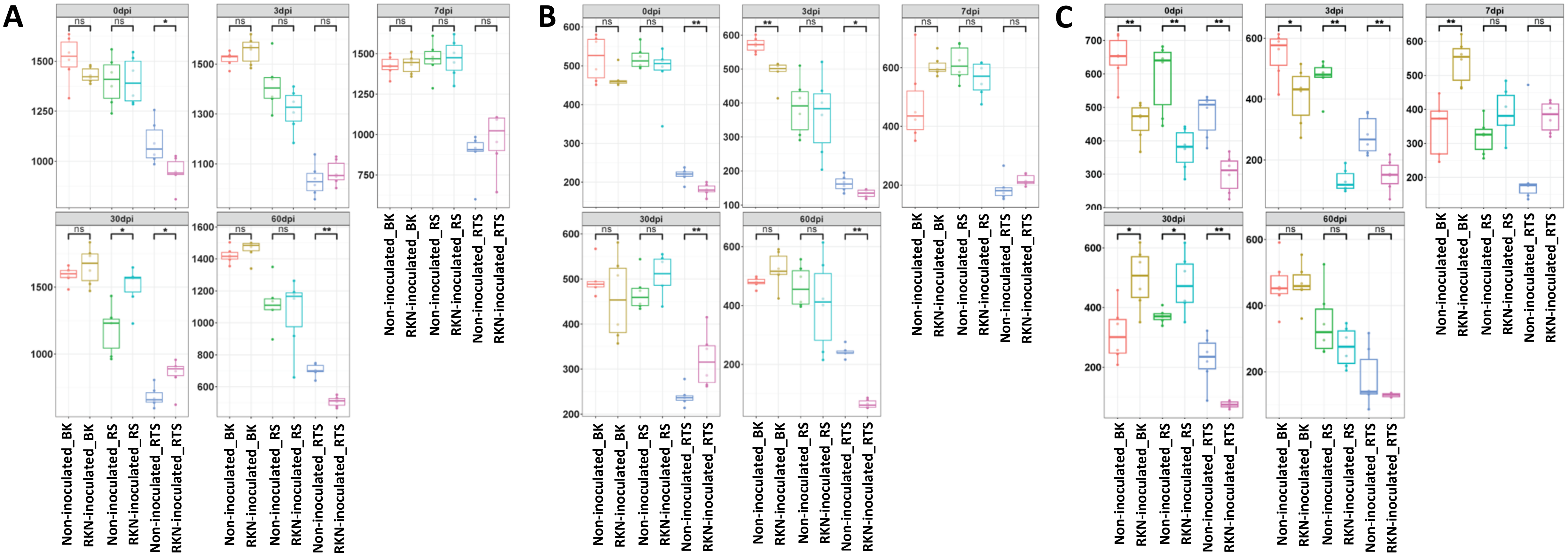

Figure S4. Observed richness of **A**) bacterial **B**) fungal and **C**) eukaryotes in RKN-inoculated and non-inoculated setups across different sampling time points

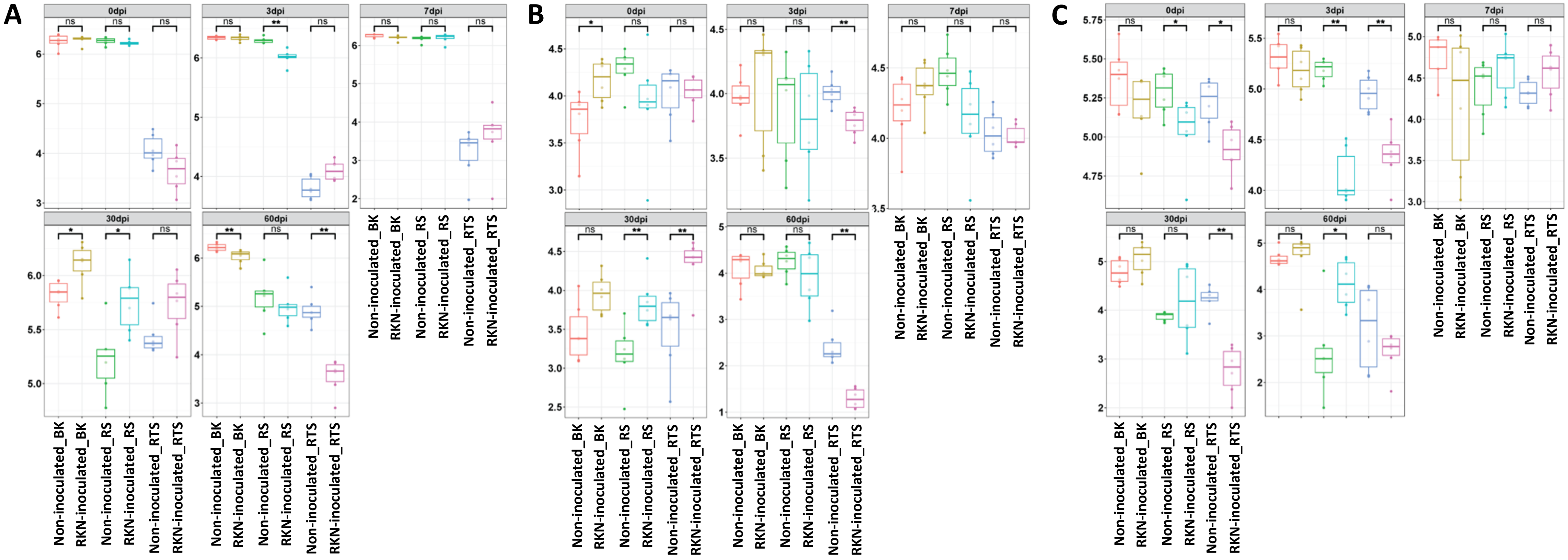

Figure S5. Shannon diversity of **A**) bacterial **B**) fungal and **C**) eukaryotes in RKN-inoculated and non-inoculated setups across different sampling time points.

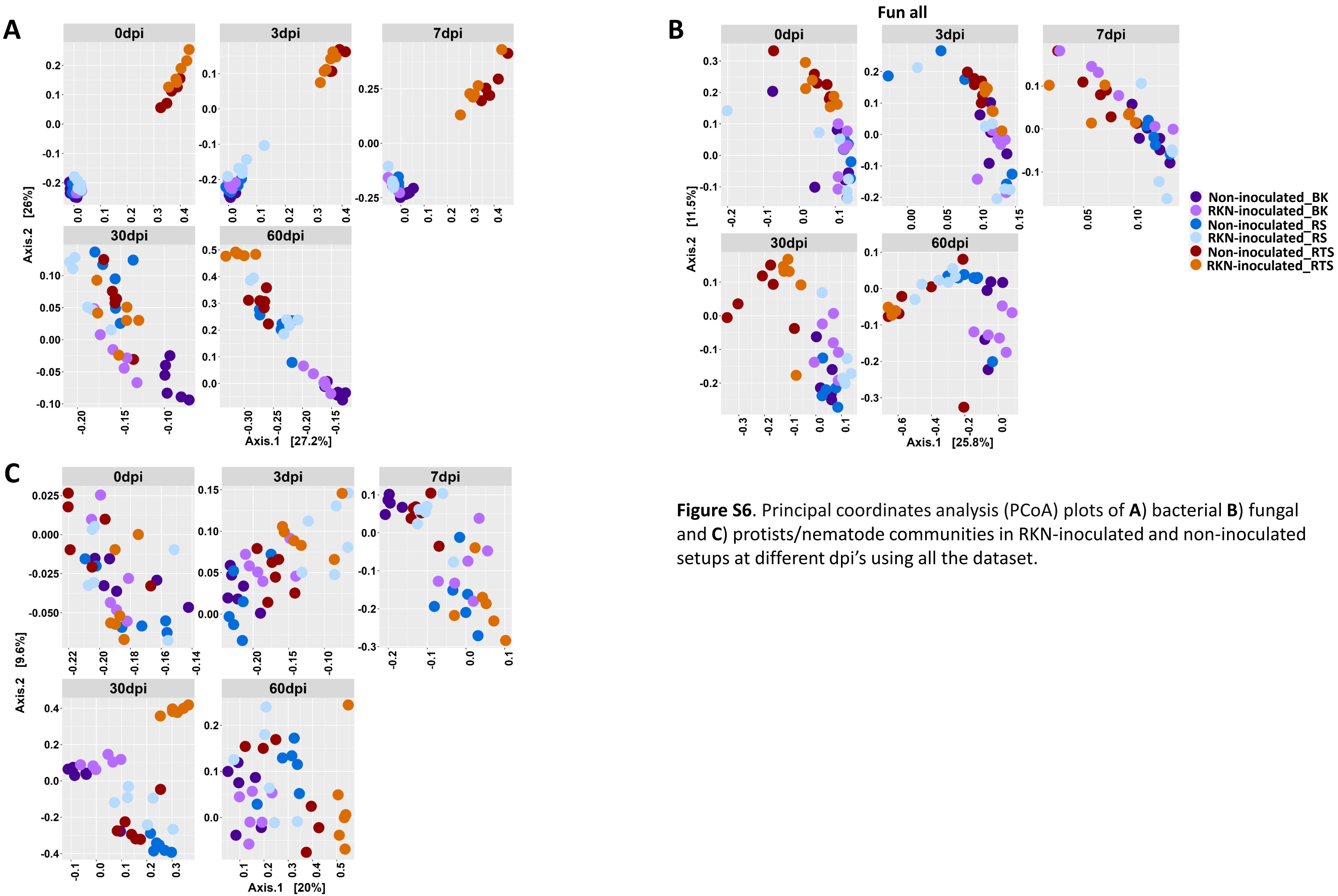

Figure S6. Principal coordinates analysis (PCoA) plots of **A**) bacterial **B**) fungal and **C**) protists/nematode communities in RKN-inoculated and non-inoculated setups at different dpi's using all the dataset.

Rhizosphere

Roots

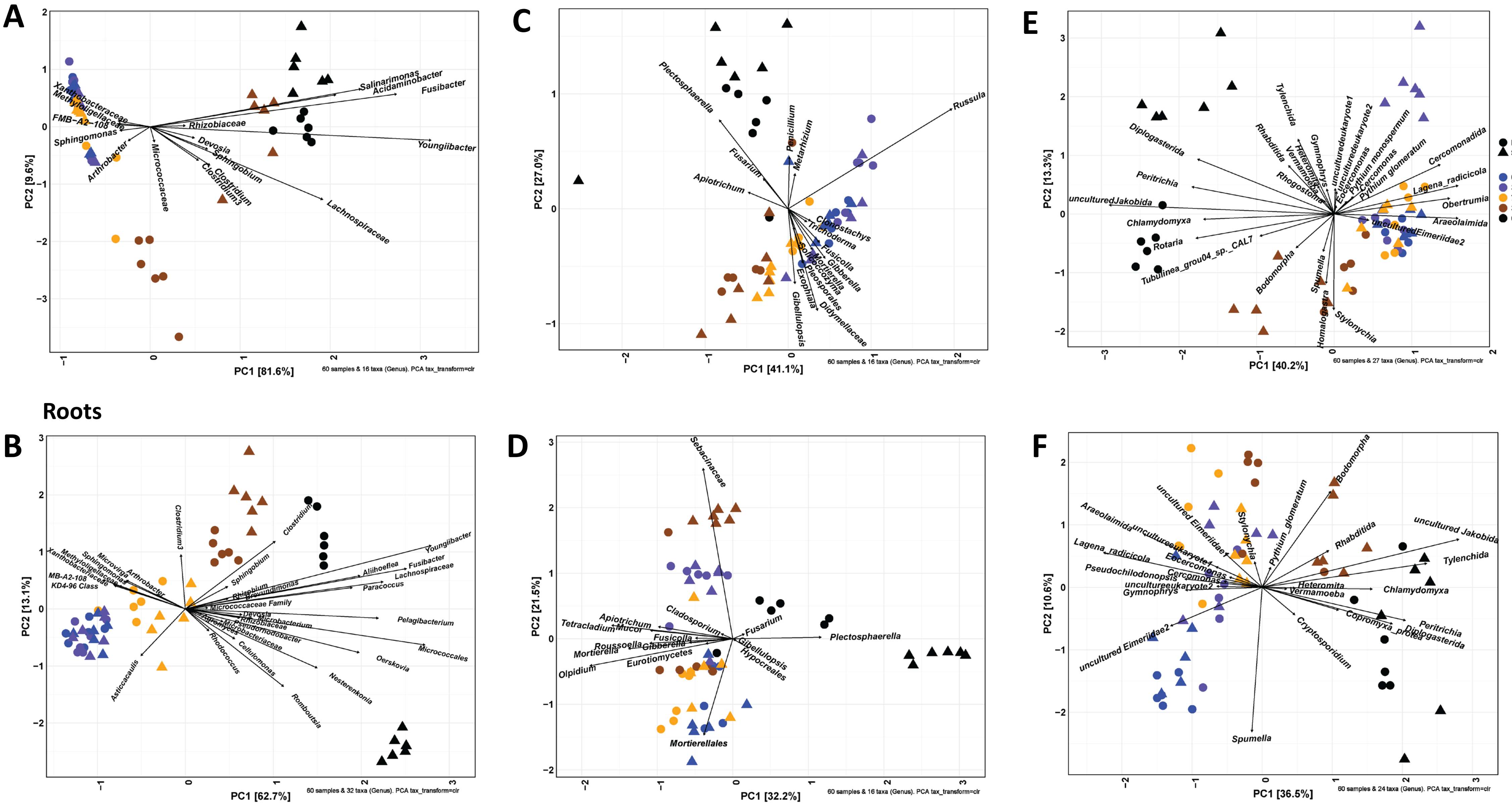

Figure S7. Unconstrained Principal Components Analysis (PCA) plots based on the Aitchison distance, showing rhizosphere and roots of bacterial (**A and B**), fungal (**C and D**) and protists/nematode (**E and F**) community compositions in RKN-inoculated and non-inoculated (controls) at different sampling time points.

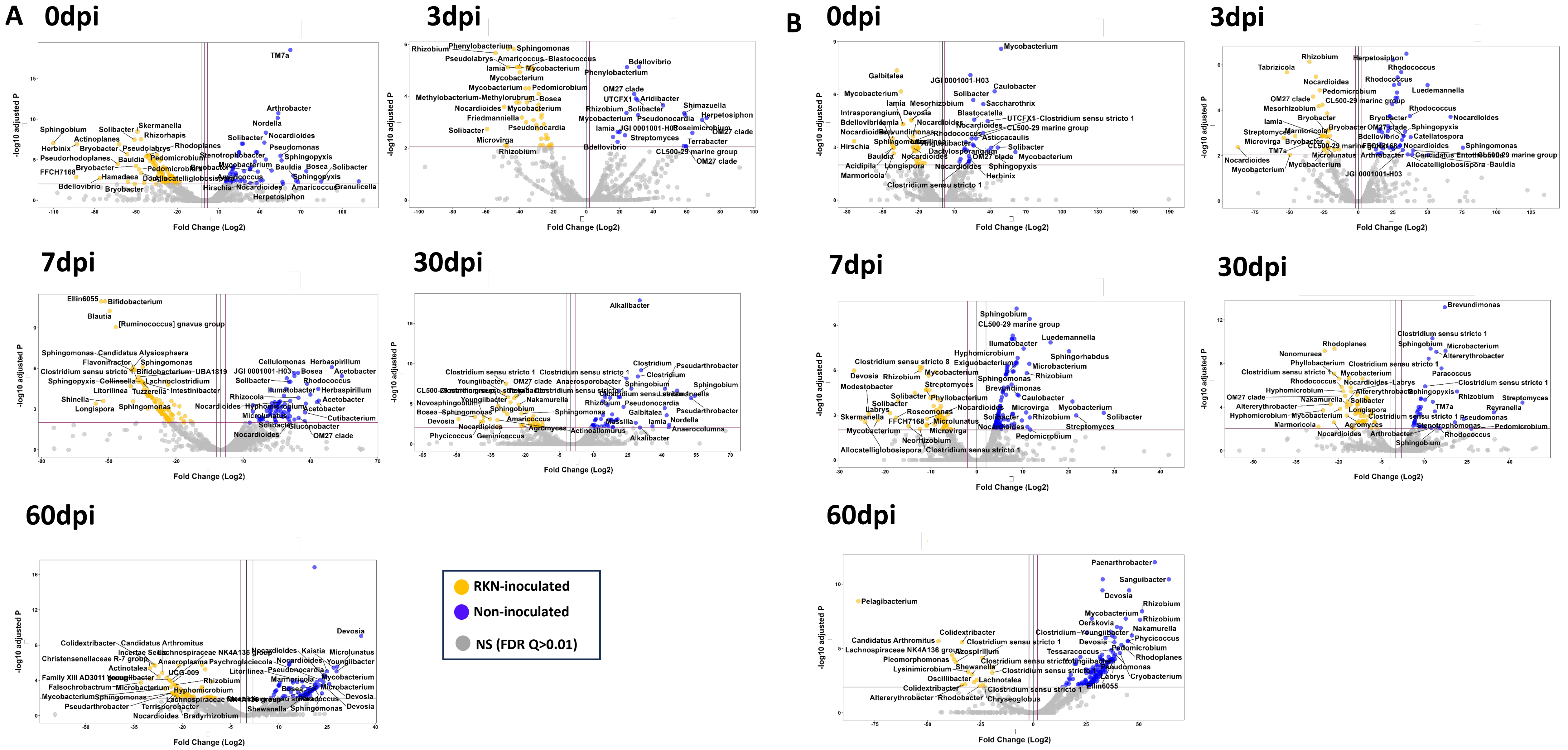

Figure S8. Volcano plot visualization of differentially abundant bacterial taxa (at ASV levels) in **A**) rhizosphere and **B**) roots of RKN-inoculated and non-inoculated samples at different dpi's. Each point represented an individual ASV assigned to corresponding taxa names. The position along the x-axis represents the direction of fold change. The red line showed the threshold of significant differential ASVs (|log2(FC)|>=2). Taxa names of 20 most differentially abundant taxa were shown in each compared set. Differentially enriched ASVs in non-inoculated and RKN-inoculated samples are shown in blue and yellow dots, respectively, while gray dots represent non-significant ASVs.

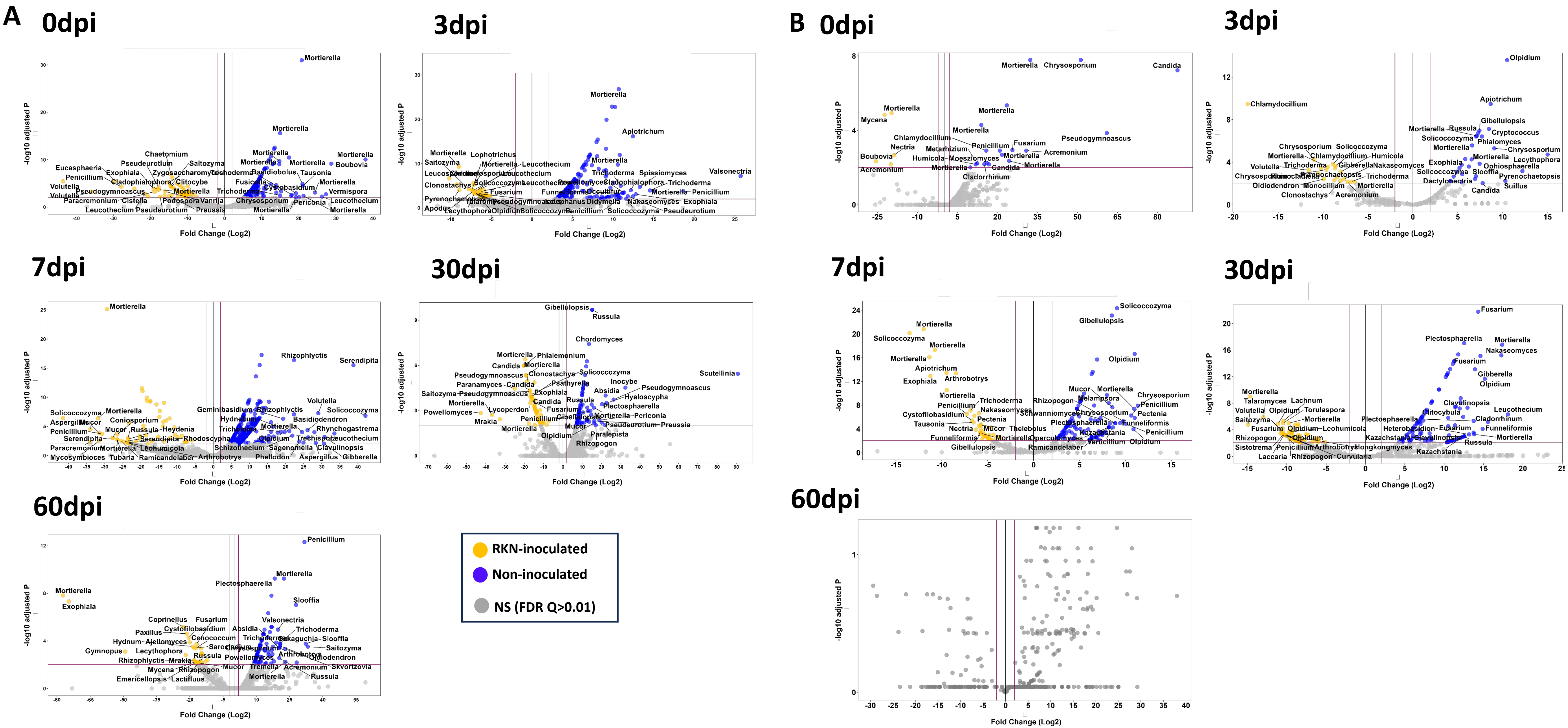

Figure S9. Volcano plot visualization of differentially abundant abundant fungal taxa (at ASV levels) in **A**) rhizosphere and **B**) root of RKN-inoculated and non-inoculated samples at different dpi's. Each point represented an individual ASV assigned to corresponding taxa names. The position along the x-axis represents the direction of fold change. The red line showed the threshold of significant differential ASVs (|log2(FC)|>=2). Taxa names of 20 most differentially abundant taxa were shown in each compared set. Differentially enriched ASVs in non-inoculated and RKN-inoculated samples are shown in blue and yellow dots, respectively , while gray dots represent non-significant ASVs.

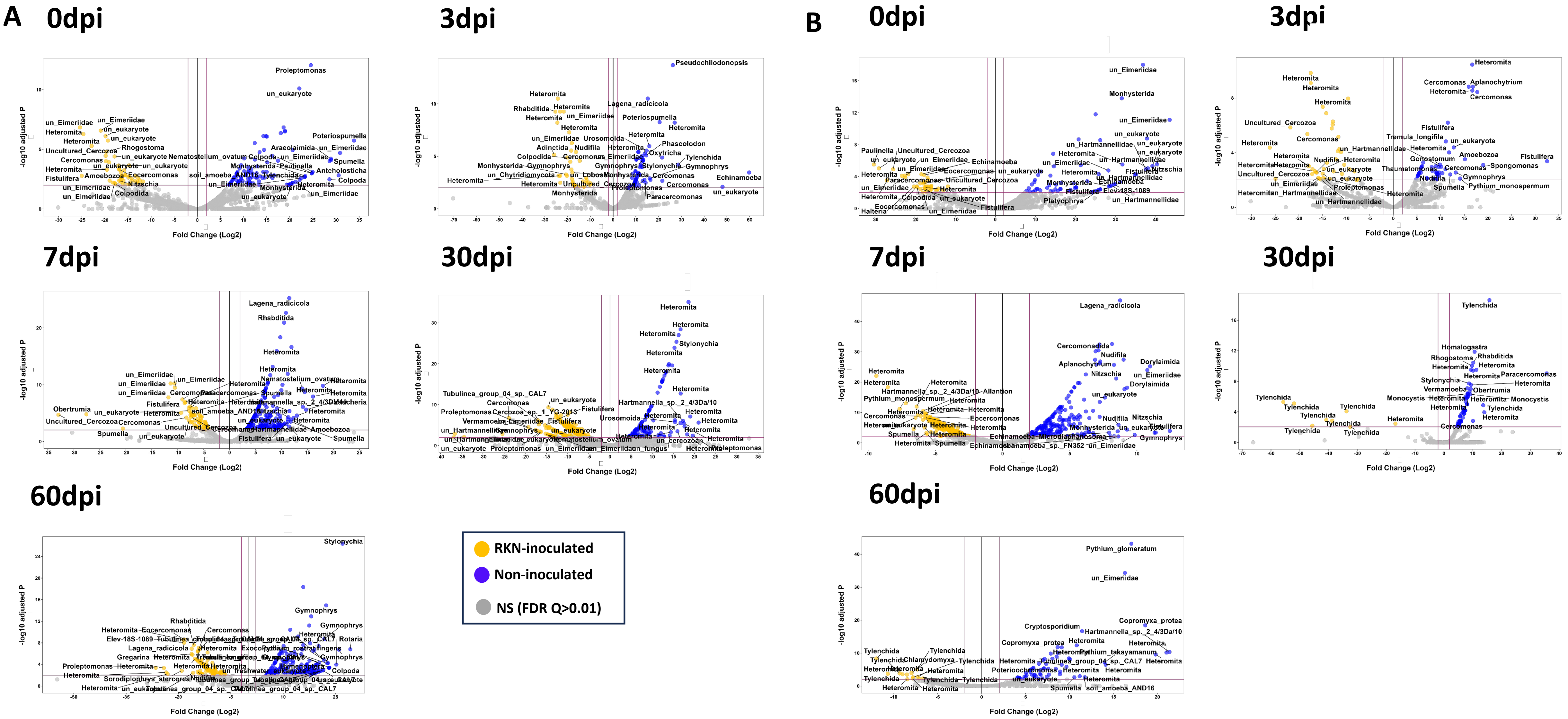

Figure S10. Volcano plot visualization of differentially abundant abundant protists/nematode taxa (at ASV levels) in **A**) rhizosphere and **B**) root of RKN-inoculated and non-inoculated samples at different dpi's. Each point represented an individual ASV assigned to corresponding taxa names. The position along the x-axis represents the direction of fold change. The red line showed the threshold of significant differential ASVs (|log2(FC)|>=2). Taxa names of 20 most differentially abundant taxa were shown in each compared set. Differentially enriched ASVs in non-inoculated and RKN-inoculated samples are shown in blue and yellow dots, respectively, while gray dots represent non-significant ASVs.

Supplementary Table S2: Mean, median and range of reads per compartment for bacterial, fungal and eukaryote libraries.

	Compartment	Mean	Median	Min	Max	Total
Bacteria (16S)	Non-inoculated	47464.1	50067.5	16125	70352	4271767
	RKN-inoculated	50851.3	50767.5	15693	79352	4576616
Fungi (ITS)	Non-inoculated	106056.5	127972.5	4168	197541	9545089
	RKN-inoculated	100277	130952	3718	182357	9024969
Eukaryotes (18S)	Non-inoculated	38163.3	29890	2834	130224	3396534
	RKN-inoculated	36657.9	20407	2042	142304	3299213

Supplementary Table S3: Percent abundance of Tylenchida in eukaryotes community and *M. incognita* in Tylenchida in samples of RKN-inoculated setups, at different sampling time points

Percent abundance of Tylenchida										
	OTU	Sample	%Abundance	Treatment	Time	SamTime	SampleType	Class	Order	
2603	ASV_47	RKNBKT13dpi	1.323733286	RKN-inoculated	T1	3dpi	BK	Chromadorea	Tylenchida	
2604	ASV_47	RKNRAST13dpi	1.390742223	RKN-inoculated	T1	3dpi	RS	Chromadorea	Tylenchida	
2610	ASV_47	RKNRTST430dpi	75.13035108	RKN-inoculated	T4	30dpi	RTS	Chromadorea	Tylenchida	
2608	ASV_47	RKNRAST560dpi	1.87012987	RKN-inoculated	T5	60dpi	RS	Chromadorea	Tylenchida	
2599	ASV_47	RKNRTST560dpi	17.05138727	RKN-inoculated	T5	60dpi	RTS	Chromadorea	Tylenchida	
Percent abundance of *M. incognita*										
	OTU	Sample	Abundance	RKN-inoculated	Time	SamTime	SampleType	Family	Genus	Species
121	ASV_47	RKNBKT0	15.51724138	RKN-inoculated	T0	3dpi	BK	Chromadorea	Tylenchida	*Meloidogyne_incognita*
123	ASV_47	RKNBKT1	13.06306306	RKN-inoculated	T1	3dpi	BK	Chromadorea	Tylenchida	*Meloidogyne_incognita*
131	ASV_47	RKNRAST1	43.90756303	RKN-inoculated	T1	3dpi	RS	Chromadorea	Tylenchida	*Meloidogyne_incognita*
127	ASV_47	RKNRTST1	80.06134969	RKN-inoculated	T1	3dpi	RTS	Chromadorea	Tylenchida	*Meloidogyne_incognita*
125	ASV_47	RKNBKT2	30.46357616	RKN-inoculated	T2	7dpi	BK	Chromadorea	Tylenchida	*Meloidogyne_incognita*
133	ASV_47	RKNRAST2	3.149606299	RKN-inoculated	T2	7dpi	RS	Chromadorea	Tylenchida	*Meloidogyne_incognita*
129	ASV_47	RKNRTST2	27.86259542	RKN-inoculated	T2	7dpi	RTS	Chromadorea	Tylenchida	*Meloidogyne_incognita*
126	ASV_47	RKNBKT4	36.58536585	RKN-inoculated	T4	30dpi	BK	Chromadorea	Tylenchida	*Meloidogyne_incognita*
122	ASV_47	RKNRAST4	4.354354354	RKN-inoculated	T4	30dpi	RS	Chromadorea	Tylenchida	*Meloidogyne_incognita*
135	ASV_47	RKNRTST4	77.10513204	RKN-inoculated	T4	30dpi	RTS	Chromadorea	Tylenchida	*Meloidogyne_incognita*
128	ASV_47	RKNBKT5	64.74719101	RKN-inoculated	T5	60dpi	BK	Chromadorea	Tylenchida	*Meloidogyne_incognita*
124	ASV_47	RKNRAST5	44.11177645	RKN-inoculated	T5	60dpi	RS	Chromadorea	Tylenchida	*Meloidogyne_incognita*
132	ASV_47	RKNRTST5	79.53939736	RKN-inoculated	T5	60dpi	RTS	Chromadorea	Tylenchida	*Meloidogyne_incognita*

Supplementary Table S4: Summary of permutational analysis of variance (PERMANOVA) using the "adonis" test on Bray-Curtis distance matrices for bacterial, fungal and eukaryotes community dissimilarity assessment using 1,000 permutations. The whole dataset including BK was used for this analysis.

**Dataset**	**Factors**	**Bacteria (16S) R^2**	**Fungi (ITS) R^2**	**Eukaryotes (18S) R^2**
Whole	Compartment	0.22***	0.35***	0.07***
	treatment	0.01*	0.01*	0.01**
	Compartment x treatment	ns	ns	0.02***
0 dpi	Compartment	0.68***	0.21***	0.10 **

	treatment	ns	0.05*	0.12***
	Compartment x treatment	ns	ns	0.17***
3 dpi	Compartment	0.68***	0.29***	0.15***
	treatment	0.03*	0.09***	0.13***
	Compartment x treatment	0.03	ns	0.13***
7 dpi	Compartment	0.70***	0.27***	0.08**
	treatment	0.04*	0.05**	0.12***
	Compartment x treatment	ns	0.08**	0.21***
30 dpi	Compartment	0.27***	0.34***	0.27 ***
	treatment	0.15***	0.07***	0.16***
	Compartment x treatment	0.07**	0.09**	0.17***
60 dpi	Compartment	0.38***	0.40***	0.23 ***
	treatment	0.16***	0.10***	0.09***
	Compartment x treatment	0.15***	0.14***	0.12***

Significance of test indicated as ***, p <0.001; **, p < 0.01; *, p <0.05. The ns denotes not statistically significant, - not determined and R^2 is the proportion of variation explained.

Supplementary Table S5: Beta diversity based pairwise comparison between compartments of RKN inoculated (RKN) and non-inoculated (Non-inoculated) samples using pairwise.adonis function

**Community**	**DPI**	**Factors (Compartment)**	**R^2 (All dataset)**	**R^2 (Without BK)**
Bacteria	T0	Non-inoculated_BK _vs_ RKN-inoculated_BK	0.12*	-
		Non-inoculated_RS _vs_ RKN-inoculated_RS	ns	ns
		Non-inoculated_RTS _vs_ RKN-inoculated_RTS	0.17*	0.18*
	T1	Non-inoculated_BK _vs_ RKN-inoculated_BK	0.22**	-
		Non-inoculated_RS _vs_ RKN-inoculated_ RS	0.17*	0.18*
		Non-inoculated_RTS _vs_ RKN-inoculated_RTS	0.20**	0.20
	T2	Non-inoculated_BK _vs_ RKN-inoculated_BK	0.32**	-

		Non-inoculated_RS _vs_ RKN-inoculated_ RS	ns	ns
		Non-inoculated_RTS _vs_ RKN-inoculated_RTS	ns	ns
	T4	Non-inoculated_BK _vs_ RKN-inoculated_BK	0.35**	-
		Non-inoculated_RS _vs_ RKN-inoculated_ RS	0.21**	0.21**
		Non-inoculated_RTS _vs_ RKN-inoculated_RTS	0.38**	0.37**
	T5	Non-inoculated_BK _vs_ RKN-inoculated_BK	0.37**	-
		Non-inoculated_RS _vs_ RKN-inoculated_ RS	0.37**	0.39**
		Non-inoculated_RTS _vs_ RKN-inoculated_RTS	0.62**	0.59**
Fungi	T0	Non-inoculated_BK _vs_ RKN-inoculated_BK	ns	-
		Non-inoculated_RS _vs_ RKN-inoculated_ RS	0.12*	0.12*
		Non-inoculated_RTS _vs_ RKN-inoculated_RTS	0.20*	0.20*
	T1	Non-inoculated_BK _vs_ RKN-inoculated_BK	0.36**	-
		Non-inoculated_RS _vs_ RKN-inoculated_ RS	ns	ns
		Non-inoculated_RTS _vs_ RKN-inoculated_RTS	0.18*	0.18
	T2	Non-inoculated_BK _vs_ RKN-inoculated_BK	0.22**	-
		Non-inoculated_RS _vs_ RKN-inoculated_ RS	ns	0.13*
		Non-inoculated_RTS _vs_ RKN-inoculated_RTS	ns	ns
	T4	Non-inoculated_BK _vs_ RKN-inoculated_BK	0.25*	-
		Non-inoculated_RS _vs_ RKN-inoculated_ RS	0.18*	0.18*
		Non-inoculated_RTS _vs_ RKN-inoculated_RTS	0.25**	0.25**
	T5	Non-inoculated_BK _vs_ RKN-inoculated_BK	0.19**	-

		Non-inoculated_RS _vs_ RKN-inoculated_ RS	0.20**	0.20**
		Non-inoculated_RTS _vs_ RKN-inoculated_RTS	0.57**	0.57**
Eukaryotes	T0	Non-inoculated_BK _vs_ RKN-inoculated_BK	0.28***	-
		Non-inoculated_RS _vs_ RKN-inoculated_ RS	0.35***	0.35**
		Non-inoculated_RTS _vs_ RKN-inoculated_RTS	0.31***	0.31**
	T1	Non-inoculated_BK _vs_ RKN-inoculated_BK	0.27**	-
		Non-inoculated_RS _vs_ RKN-inoculated_RS	0.40**	0.40**
		Non-inoculated_RTS _vs_ RKN-inoculated_RTS	0.23**	0.23**
	T2	Non-inoculated_BK _vs_ RKN-inoculated_BK	0.47**	-
		Non-inoculated_RS _vs_ RKN-inoculated_RS	0.25**	0.25**
		Non-inoculated_RTS _vs_ RKN-inoculated_RTS	0.36**	0.36***
	T4	Non-inoculated_BK _vs_ RKN-inoculated_BK	0.26**	-
		Non-inoculated_RS _vs_ RKN-inoculated_RS	0.26**	0.26**
		Non-inoculated_RTS _vs_ RKN-inoculated_RTS	0.70**	0.70**
	T5	Non-inoculated_BK _vs_ RKN-inoculated_BK	0.24**	-
		Non-inoculated_RS _vs_ RKN-inoculated_RS	0.29**	0.29**
		Non-inoculated_RTS _vs_ RKN-inoculated_RTS	0.26**	0.26**

Significance of test indicated as ***, p <0.001; **, p < 0.01; *, p <0.05. The ns denotes not statistically significant, - not determined and R^2 is the proportion of variation explained.
